# ﻿Abyssal fauna of polymetallic nodule exploration areas, eastern Clarion-Clipperton Zone, central Pacific Ocean: Amphinomidae and Euphrosinidae (Annelida, Amphinomida)

**DOI:** 10.3897/zookeys.1137.86150

**Published:** 2022-12-22

**Authors:** Lenka Neal, Helena Wiklund, Laetitia M. Gunton, Muriel Rabone, Guadalupe Bribiesca-Contreras, Thomas G. Dahlgren, Adrian G. Glover

**Affiliations:** 1 Life Sciences Department, Natural History Museum, London SW7 5BD, UK Life Sciences Department, Natural History Museum London United Kingdom; 2 Department of Marine Sciences, University of Gothenburg, Box 463, 40530 Gothenburg, Sweden University of Gothenburg Gothenburg Sweden; 3 Gothenburg Global Biodiversity Centre, Box 463, 40530 Gothenburg, Sweden Gothenburg Global Biodiversity Centre Gothenburg Sweden; 4 Australian Museum Research Institute, 1 William Street, Sydney NSW 2010, Australia Australian Museum Research Institute Sydney Australia; 5 NORCE Norwegian Research Centre, Bergen, Norway NORCE Norwegian Research Centre Bergen Norway

**Keywords:** Amphinomida, CCZ, COI, deep-sea mining, molecular phylogeny, species distribution, taxonomic novelty, 18S, 16S

## Abstract

This is a contribution in a series of taxonomic publications on benthic fauna of polymetallic nodule fields in the eastern abyssal Clarion-Clipperton Zone (CCZ). The material was collected during environmental surveys targeting exploration contract areas ‘UK-1’, ‘OMS’ and ‘NORI-D’, as well as an Area of Particular Environmental Interest, ‘APEI-6’. The annelid families Amphinomidae and Euphrosinidae are investigated here. Taxonomic data are presented for six species from 41 CCZ-collected specimens as identified by a combination of morphological and genetic approaches; of the six species, three are here described as new, one species is likely to be new but in too poor condition to be formalised and the two others likely belong to known species. Description of three new species *Euphrosinellageorgievae***sp. nov.**, *Euphrosinopsisahearni***sp. nov.**, and *Euphrosinopsishalli***sp. nov.** increases the number of formally described new annelid species from the targeted areas to 21 and CCZ-wide to 52. Molecular data suggest that four of the species reported here are known from CCZ only, but within CCZ they have a wide distribution. In contrast, the species identified as Bathychloeiacf.sibogae Horst, 1910 was found to have a wide distribution within the Pacific based on both morphological and molecular data, using comparative material from the abyssal South Pacific. Bathychloeiacf.balloniformis Böggemann, 2009 was found to be restricted to APEI-6 based on DNA data available from CCZ specimens only, but morphological data from other locations suggest potentially a wide abyssal distribution. The genus *Euphrosinopsis* was previously known only from Antarctic waters, and *Euphrosinellageorgievae***sp. nov.** was recovered as a sister taxon to the Antarctic specimens of Euphrosinellacf.cirratoformis in our molecular phylogenetic analysis, strengthening the hypothesised link between the deep-sea and Antarctic benthic fauna.

## ﻿Introduction

The Clarion-Clipperton Zone (CCZ) polymetallic nodule region, a vast area (ca. 6 million km^2^) of the central abyssal Pacific, has been explored in recent decades for its deep-sea mineral resources and their potential for commercial mining (e.g., Gollner et al. 2017; Glover et al. 2018; Smith et al. 2021). Such exploration is managed through exploration licenses, regulated by the International Seabed Authority (ISA), which stipulates the need for biodiversity baseline studies, environmental impact assessments and the establishment of preservation areas (Lodge et al. 2014; Washburn et al. 2021). This paper is based on material collected within areas of the eastern CCZ prospected by 1) the UK Seabed Resources Ltd (UKSRL), exploration contract area ‘UK-1’, 2) the Ocean Mineral Singapore exploration contract area ‘OMS’, and 3) Nauru Ocean Resources Inc (NORI), exploration contract area ‘NORI-D’. Additional material studied was collected from the Area of Particular Environmental Interest ‘APEI-6’.

The knowledge of the biodiversity and distribution of benthic taxa found within areas of potential mining operations is paramount to informed environmental impact assessments and conservation efforts (Smith et al. 2011, 2021). Both biodiversity and species ranges remain poorly understood within this area mainly due to under-sampling and the lack of comparable datasets produced by different research groups and contractors. The latter factor is greatly confounded by the lack of formal descriptions of the fauna given that most represent species new to science (Glover et al. 2018). This lack of knowledge is particularly acute for sediment infauna, a benthic component of fauna that cannot be captured by video or camera surveys. In general, annelids dominate the abyssal sediment macrofauna, constituting 50–75% of macrofaunal abundance and species richness, and are therefore considered a key component of benthic biodiversity (e.g., Glover et al. 2002; Smith et al. 2008). Annelids also exhibit a broad range of feeding types and life-history strategies and are frequently used to evaluate anthropogenic disturbance in shallow-water habitats (Dean 2008). Thus, evaluation of the diversity and species ranges of annelids is critical to predicting and managing the impacts of proposed nodule mining in the CCZ.

Our main objective has been to provide taxonomic hypotheses on macrofaunal annelids collected from the targeted areas within the CCZ based on morphology and molecular data. These data build up on previous taxonomic work on annelids from the target areas (Wiklund et al. 2019; Drennan et al. 2021; Neal et al. 2022) as well as wider CCZ area (Janssen et al. 2015; Blake 2019; Bonifácio and Menot 2019) and ultimately provide further insights into annelid species distribution in the deep-sea realm. Up to this date, 52 annelid species have been formally described from CCZ, with the focus on families Spionidae (Paterson et al. 2016; Neal et al. 2022), Orbiniidae (Blake 2017, 2020), Polynoidae (Bonifácio and Menot 2019), Cirratulidae (Blake 2016, 2019), Opheliidae, Scalibregmatidae, and Travisiidae (Wiklund et al. 2019), Syllidae (Maciolek 2020) and Nereididae (Drennan et al. 2021).

Annelids of order Amphinomida are commonly known as fire worms due to the skin burning sensation upon contact with their chaetae caused by a complex mixture of defensive toxins (Verdes et al. 2018), although the exact nature of the toxin delivery is still a matter of debate (Righi et al. 2021a, b; Tilic and Bartolomaeus 2021). Most Amphinomida species are carnivores or scavengers, but some may also ingest detritus and algae (Fauchald and Jumars 1979; Jumars et al. 2015). Amphinomida has a widespread distribution, occurring from the intertidal to deep waters, with good representation in extreme environments such as the Antarctic shelf (Kudenov 1993), chemosynthetic habitats (e.g., Fiege and Bock 2009; Borda et al. 2013; Barroso et al. 2018, 2021), hypoxic environments (Jeffreys et al. 2012) and polluted sites such as fish farms (LN pers. obs.). One species even inhabits the lumen of the digestive tract of a deep-sea spatangoid (Emson et al. 1993).

In terms of their systematics, Amphinomida were regarded as part of the Errantia (e.g., Rouse and Fauchald 1997) until molecular studies revealed their phylogenetic position basally within Annelida and as a sister group to sipunculids (e.g., Weigert et al. 2014). Such relationship with the unsegmented and sessile worms is hard to explain based only on morphology as no synapomorphies have been found to date (Beckers and Tilic 2021). Currently, the order Amphinomida contains two accepted families, Amphinomidae Lamarck, 1818 and Euphrosinidae Williams, 1852 (Read and Fauchald 2021). As a result of recent molecular phylogenetic analyses (Wiklund et al. 2008; Borda et al. 2015) Amphinomidae have been further divided into two subfamilies Amphinominae Lamarck, 1818 and Archinominae Kudenov, 1991. Amphinomidae has 180 species belonging to 22 genera, with the bulk of Amphinominae living in shallow warm and temperate waters, while members of Archinominae tend to inhabit deep-sea extreme habitats. The less diverse Euphrosinidae contains approximately 60 accepted species belonging to only four genera (Read and Fauchald 2021). Most of euphrosinid diversity lies within the geographically widespread genus *Euphrosine* Lamarck, 1818 with 54 species, while the small and rarely encountered genera *Palmyreuphrosyne* Fauvel, 1913; *Euphrosinella* Detinova, 1985 and *Euphrosinopsis* Kudenov, 1993 tend to be confined to the deep-sea and the Antarctic shelf (Kudenov 1993).

## ﻿Materials and methods

### ﻿Fieldwork

**Figure 1.**
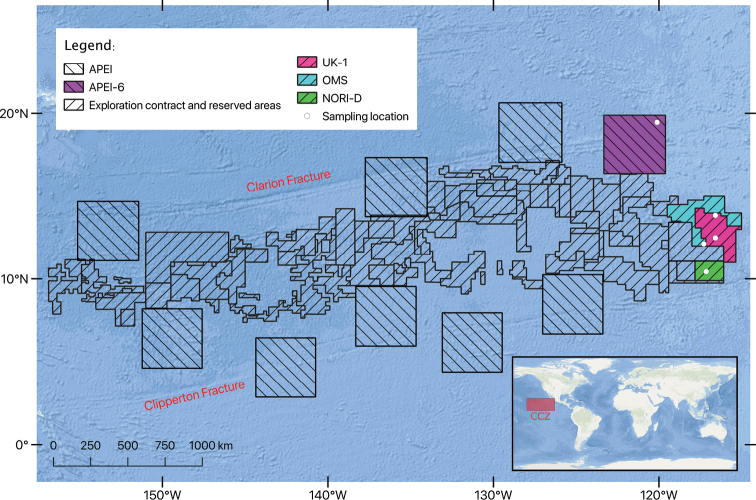
Map of CCZ of exploration areas and areas of particular interest, with targeted areas where samples of this study were collected highlighted in colours (see legend for explanation).

The first UKSR ABYSSLINE cruise (AB01) took place in October 2013 onboard the RV ‘Melville’ and targeted the UK-1 exploration contract area (Fig. 1). The second cruise (AB02) took place in February–March 2015 onboard RV ‘Thomas G. Thompson’ and sampled a wider area (Fig. 1), including: UK-1 (depth ca. 4200 m) and OMS (depth ca. 4200 m) exploration contract areas and APEI-6 (depth ca. 4050 m), an area exempted from mining activities (Wedding et al. 2013). The Resource Cruise 01 (RC01) took place aboard the marine vessel M/V ‘Pacific Constructor’ between February and March 2020 and targeted exploration contract areas UK-1 and OMS (Fig. 1). Nauru Ocean Resources Inc (**NORI)** Campaign 05a (DG05a) cruise took place between October and November 2020 and the 05d (DG05d) cruise took place between April and June 2021, both expeditions were onboard ‘Maersk Launcher’ to the NORI-D exploration contract area (depth ca. 4300 m) (Fig. 1).

For a comprehensive description of the methodological pipeline, see Glover et al. (2016b). Briefly, specimens were collected using box corer and Brenke epibenthic sledge (EBS) (Brenke 2005). Geographic data from sampling activities were recorded on a central GIS database (Fig. 1). Live-sorting of specimen samples was carried out onboard all four vessels in a ‘cold-chain’ pipeline, with material maintained in chilled (2–4 °C), filtered seawater. Specimens were assigned preliminarily identification and imaged live using stereo microscopes with attached digital cameras (Glover et al. 2016b). Specimens were then stored in individual microtube vials filled with aqueous solution of 80% non-denatured ethanol labelled appropriately and entered into a local database. Samples were kept chilled throughout their transportation to the Natural History Museum, London, UK (**NHMUK**).

### ﻿Morphological laboratory work

In the laboratory, preserved specimens were re-examined using stereo and compound microscopes. They were identified to morphospecies, and the best-preserved examples (voucher specimens) were then used to provide informal descriptions with key morphological features photographed with digital camera. Shirlastain A was used during the morphological examination on some specimens, in order to better observe certain characters. Scanning electron microscopy (**SEM**) using a SEM FEI Quanta 650 was conducted on selected specimens, following graded ethanol dehydration, critical point drying, and gold coating. Figures were assembled using Adobe Photoshop CS6 software. In some instances, a fine line was used to outline and highlight particular morphological features where such features were unclear from images alone. Line drawings were made using camera lucida system.

Additionally, Amphinomidae specimens recently collected from the abyssal South Pacific (ca. 4000 m) as part of the RV ‘Investigator’ voyage ‘Sampling the Abyss’ were made available for examination (see also Gunton et al. 2021). These specimens were examined as described above. Material registered and lodged at the Australian Museum is prefixed (AM W.).

### ﻿Molecular laboratory work

Extraction of DNA was done with DNeasy Blood and Tissue Kit (Qiagen) using a Hamilton Microlab STAR Robotic Workstation. Approximately 1800 bp of 18S were amplified using the primers 18SA 5’-AYCTGGTTGATCCTGCCAGT-3’(Medlin et al. 1988) and 18SB 5’-ACCTTGTTACGACTTTTACTTCCTC-3’ (Nygren and Sundberg 2003), ca. 450 bp of 16S were amplified with the primers ann16Sf 5’-GCGGTATCCTGACCGTRCWAAGGTA-3’ (Sjölin et al. 2005) and 16SbrH 5’-CCGGTCTGAACTCAGATCACGT-3’ (Palumbi 1996), and ca. 650 bp of cytochrome *c* oxidase were amplified using LCO1490 5’-GGTCAACAAATCATAAAGATATTGG-3’ (Folmer et al. 1994) and COI-E 5’-TATACTTCTGGGTGTCCGAAGAATCA-3’ (Bely and Wray 2004). PCR mixtures contained 1 µl of each primer (10 µM), 2 µl template DNA and 21 µl of Red Taq DNA Polymerase 1.1X MasterMix (VWR) in a mixture of total 25 µl. The PCR amplification profile for all gene fragments consisted of initial denaturation at 95 °C for 5 min, 35 cycles of denaturation at 94 °C for 45 s, annealing at 55 °C for 45 s, extension at 72 °C for 2 min, and a final extension at 72 °C for 10 min. PCR products were purified using Millipore Multiscreen 96-well PCR Purification System, and sequencing was performed on an ABI 3730XL DNA Analyser (Applied Biosystems) at The Natural History Museum Sequencing Facility, using the same primers as in the PCR reactions plus two internal primers for 18S, 620F 5’-TAAAGYTGYTGCAGTTAAA-3’ (Nygren and Sundberg 2003) and 1324R 5’-CGGCCATGCACCACC-3’ (Cohen et al. 1998). Overlapping sequence fragments were merged into consensus sequences using Geneious (Kearse et al. 2012) and aligned using MAFFT (Katoh et al. 2002) for 18S and 16S, and MUSCLE (Edgar 2004) for COI, both programs used as plugins in Geneious, with default settings.

Molecular data were used to place species covered in this study within the Amphinomida phylogenetic relationships. The phylogenetic analyses were done in two parts, producing one tree for Euphrosinidae with a taxon from Amphinomidae as root, and one for Amphinomidae with a taxon from Euphrosinidae as root. Sequences added from GenBank are listed in Supplementary data with taxon names and sequence accession numbers. The program jModelTest (Posada 2008) was used to assess the best model for each partition with BIC, which suggested the for MrBayes possible GTR+I+G as the best model for all genes. The data was partitioned into two genes (18S and 16S) for Euphrosinidae and three genes (18S, 16S and COI) for Amphinomidae, and the evolutionary model mentioned above was applied to each partition. The parameters used for the partitions were unlinked. Bayesian phylogenetic analyses (BAs) were conducted with MrBayes v. 3.2.6 (Ronquist et al. 2012). Analyses were run three times for 10,000,000 generations. Of these, the first 2,500,000 generations were discarded as burn-in. The tree files were interpreted with FigTree ver. 1.4.4 (available from http://tree.bio.ed.ac.uk/software/figtree/). Uncorrected ‘p’ for one of the species, Bathychloeiacf.sibogae was calculated from a COI alignment of 534 characters using Mesquite (Maddison and Maddison 2021).

### ﻿Taxonomic assignments

Here we use a phylogenetic species concept *sensu*Donoghue (1985) with species determined by DNA-based phylogenetic analysis. The poor morphological preservation and the subsequent lack of morphological data did not always allow for formal description of a new species. Instead, we provide the lowest-level taxonomic name possible aided by phylogenetic information. In these cases, we use an informal naming system where the voucher specimen number is used as the informal species name. Therefore, *Paramphinome* sp. NHM_6022E is the informal species name for all specimens that belong to the same species as the specimen number NHM_6022E. This avoids confusion with the use of sp. A, sp. B, sp. C etc. where informal and confusing synonyms can easily arise. Newly formalised species were named in honour of the scientists, technicians, and crew of the vessels used during the CCZ cruises reported here, with the names being selected from a randomised list from all on board. Type material, DNA specimen vouchers and DNA extractions are deposited at the Natural History Museum, London. A full list of all taxa including Natural History Museum Accession Numbers (NHMUK), NHM Molecular Collection Facility (NHM-MCf), and NCBI GenBank accession numbers are provided in Table 1.

**Table 1.** List of taxa presented in this paper – family, DNA taxonomy ID (a species-level identification based on combined DNA and morphological evidence), cruise record number, GUID (Global Unique Identifier link to data record at http://data.nhm.ac.uk), NHMUK registration number (NHMUK), Molecular Collection facility (MCf) sample ID number (MCF no.) and NCBI GenBank accession numbers (COI/16S/18S AK no.) for successfully sequenced genetic markers. GenBank numbers for phylogenetic analysis data downloaded from GenBank are presented in Suppl. material 1.

DNA taxonomy ID	NHM no.	GUID	Reg no. NHMUK	MCf no.	COI AK no.	16S AK no.	18S AK no.
**Family Amphinomidae**							
Bathychloeiacf.balloniformis	NHM_2107	c79b4600-e8e9-4484-b06a-e18330a1421d	ANEA 2022.630	0118302190	ON903198	ON900088	ON905671
Bathychloeiacf.balloniformis	NHM_2109	ac3dd714-64ac-44ea-9168-22437dc3cfba	ANEA 2022.631	0118302189		ON900113	
Bathychloeiacf.sibogae juvenile	NHM_6880_HW01	06f82805-e608-4715-af62-ab1d44df2a79	ANEA 2022.632	0118302159	ON903200	ON900089	
Bathychloeiacf.sibogae	NHM_0821	73a7200a-ae19-4c0c-8381-8d4509a318cf	ANEA 2022.633	0118302202	ON903197	ON900100	ON905670
Bathychloeiacf.sibogae	NHM_2906	d3848fcf-4cb2-49fd-b49c-e09422419a70	ANEA 2022.634	0118302177		ON900116	
Bathychloeiacf.sibogae juvenile	NHM_2115	2cbc0d92-247c-4197-bd7a-4715adb5e8f4	ANEA 2022.635	0118302188		ON900114	
Bathychloeiacf.sibogae	NHM_3539	083df63d-60e7-48ae-95c4-6a11a61b01e8	ANEA 2022.636	0118302158	ON903199	ON900118	
Bathychloeiacf.sibogae	NHM_8922	805f34aa-ec4f-4318-b18b-46447350aa1e	ANEA 2022.637	0118302156	ON903201		
*Paramphinome* sp. NHM_6022E	NHM_1167D	fd4902df-aef2-44cf-991f-31905434c2a1	ANEA 2022.638				
*Paramphinome* sp. NHM_6022E	NHM_4044	56235559-3f2c-426e-b4cd-37462593a4ba	ANEA 2022.639	0118302160			
*Paramphinome* sp. NHM_6022E	NHM_6022E	bd4b405d-3e56-4671-909e-fdf9c3e7fbcf	ANEA 2022.640	0118302162		ON900125	ON905673
**Family Euphrosinidae**							
*Euphrosinopsishalli* sp. nov.	NHM_0779	1a683870-d904-4c2c-bf1a-a34ead0a42fc	ANEA 2022.641	0118302182		ON900099	
*Euphrosinopsishalli* sp. nov.	NHM_4339 (holotype)	670dfd34-338d-4edc-8856-b0a9a728efc9	ANEA 2022.642	0118302157		ON900119	ON905672
*Euphrosinopsishalli* sp. nov.	NHM_6018 (paratype)	ab26e2ea-ab87-4013-8106-e817c0485cc9	ANEA 2022.643	0118302167		ON900124	
*Euphrosinopsisahearni* sp. nov.	NHM_0095	a351cb41-736c-4390-8ad8-02c0358b73e0	ANEA 2022.644	0118302201		ON900092	ON905668
*Euphrosinopsisahearni* sp. nov.	NHM_0888	4d76b4e2-569d-4a17-9276-3ce721cbdf72	ANEA 2022.645	0118302187		ON900101	
*Euphrosinopsisahearni* sp. nov	NHM_0551 (paratype, SEM)	241b828d-a574-47f2-995d-0bdef239c427	ANEA 2022.646	0118302186		ON900094	
*Euphrosinopsisahearni* sp. nov	NHM_5042	1662fd8b-54a5-4f97-9083-02dbb2df7e39	ANEA 2022.647	0118302178		ON900121	
*Euphrosinopsisahearni* sp. nov.	NHM_1737A	4f372c07-c466-4b6c-91a9-229cd7c7a17d	ANEA 2022.648	0118302171		ON900107	
*Euphrosinopsisahearni* sp. nov.	NHM_1876	6ad5c2b3-ece8-4195-a19f-3913de511e71	ANEA 2022.649	0118302175		ON900112	
*Euphrosinopsisahearni* sp. nov.	NHM_0550	92791783-35c2-4fbf-80b0-2b074ef70828	ANEA 2022.650	0118302203		ON900093	
*Euphrosinopsisahearni* sp. nov.	NHM_1302	7aabe644-2ec6-4671-8c1a-f826eeeb0b46	ANEA 2022.651	0118302168		ON900105	
*Euphrosinopsisahearni* sp. nov.	NHM_1302A (holotype)	479933d3-9943-4d87-a1b8-ea120bd8f4ee	ANEA 2022.652	0118302169		ON900104	
*Euphrosinopsisahearni* sp. nov.	NHM_1737	2ca3e584-a68d-4ea5-98d2-75ce10515386	ANEA 2022.653	0118302173		ON900110	
*Euphrosinopsisahearni* sp. nov.	NHM_1737C (paratype)	efe95a8c-fc88-4849-ad26-1df3d292ef20	ANEA 2022.654	0118302172		ON900109	
*Euphrosinopsisahearni* sp. nov.	NHM_ 0616	4758bf19-c6d0-42e0-b5ba-e83e203d2e18	ANEA 2022.655	0118302185		ON900096	
*Euphrosinopsisahearni* sp. nov.	NHM_0759	b0f9162f-a861-4eb2-89a1-ce25c2bd09c4	ANEA 2022.656	0118302184		ON900097	
*Euphrosinopsisahearni* sp. nov.	NHM_1839	02a5ace7-841e-4f50-bf03-57ba21f02f7c	ANEA 2022.657	0118302174		ON900111	
*Euphrosinellageorgievae* sp. nov.	NHM_0587	b7a0bf33-0dc4-4f61-90de-35865647a99f	ANEA 2022.658	0118302191		ON900095	ON905669
*Euphrosinellageorgievae* sp. nov.	NHM_0777	a8f0e776-d7b6-4ec6-a549-78f40f17d89b	ANEA 2022.659	0118302183		ON900098	
*Euphrosinellageorgievae* sp. nov.	NHM_1737B	2784df45-eec0-4151-b12d-11d955985faa	ANEA 2022.660			ON900108	
*Euphrosinellageorgievae* sp. nov.	NHM_0910	05dfb32c-fc3a-4028-bf09-3eb840175661	ANEA 2022.661	0118302181		ON900102	
*Euphrosinellageorgievae* sp. nov.	NHM_1134 (paratype)	00590d2b-f952-4c69-8bc2-ac2a408da17a	ANEA 2022.662	0118302180		ON900103	
*Euphrosinellageorgievae* sp. nov.	NHM_1514	96cb7b69-c0ea-4559-9b57-3abe6af4a4c7	ANEA 2022.663	0118302170		ON900106	
*Euphrosinellageorgievae* sp. nov.	NHM_2391 (holotype)	1ce8325f-74de-47de-a776-2dc50b8d69ae	ANEA 2022.664	0118302176		ON900115	
*Euphrosinellageorgievae* sp. nov.	NHM_4975	677b7d67-d9cc-4ebd-8d79-cf5da5dc40da	ANEA 2022.665	0118302165		ON900120	
*Euphrosinellageorgievae* sp. nov.	NHM_6087	eebfaecd-5ee2-49d6-be73-51eb91678487	ANEA 2022.666	0118302166		ON900126	
*Euphrosinellageorgievae* sp. nov.	NHM_5802	c0e408e3-91e7-408f-aaef-3be86507105a	ANEA 2022.667	0118302164		ON900123	
*Euphrosinellageorgievae* sp. nov.	NHM_5057	d92b1574-eccb-443c-a15d-b79357360b59	ANEA 2022.668	0118302179		ON900122	
*Euphrosinellageorgievae* sp. nov.	NHM_7235	55637dc0-f9b9-4586-9bfb-7a821c785279	ANEA 2022.669	0118302163		ON900127	
*Euphrosinellageorgievae* sp. nov.	NHM_2908	fba3fab7-ae4b-4415-a73c-a2ba6cd44601	ANEA 2022.670	0118302161		ON900117	

### ﻿Data handling

The field and laboratory work led to a series of databases and sample sets that were integrated into a ‘data-management pipeline’. This included the transfer and management of data and samples between a central collections database, a molecular collections database and external repositories (GenBank, WoRMS, OBIS, GBIF, GGBN, ZooBank) through DarwinCore archives (Suppl. material 1). This provides a robust data framework to support DNA taxonomy, in which openly available data and voucher material are key to quality data standards. A further elaboration of the data pipeline is published in Glover et al. (2016b).

## ﻿Systematics section

**Amphinomidae Lamarck, 1818**

### Archinominae Kudenov, 1991

#### ﻿ Bathychloeia

Taxon classificationAnimaliaAmphinomidaAmphinomidae

Horst, 1910

9CACD744-B1EC-5B2F-9622-E3DF87C6F1E5

##### Type species.

*Bathychloeiasibogae* Horst, 1910.

##### Diagnosis

(modified from Böggemann (2009)). Body small, fusiform. Prostomium divided into an anterior and posterior lobe, with a median antenna on posterior lobe; paired lateral antennae and palps on anterior lobe. Eyes present or absent. Caruncle with well-developed folds and crenulations. Branchiae bipinnate from chaetiger 5 or 6, where enlarged. Dorsal, lateral and ventral cirri cirriform. Chaetae bifurcate. Pygidial cirri paired, cirriform to digitiform.

##### Remarks.

As the name *Bathychloeia* suggests, this genus was established for deep-water representatives similar to forms in predominantly shallow water genus *Chloeia* Lamarck, 1818. *Chloeia* was established by Lamarck (1818) to accommodate *Chloeiaflava* described from the Indian Ocean by Pallas in 1766 and currently contains 20 species occurring in the Indian, Pacific, and Atlantic oceans (Hartman 1959; Barroso and Paiva 2011). This genus is morphologically characterised by fusiform body shape and bipinnate branchiae. However, such characteristics are also shared with the rare, uniquely deep-sea genera *Bathychloeia* Horst, 1910 and *Chloenopsis* Fauchald, 1977. *Bathychloeia* has been distinguished from *Chloeia* by Horst (1910, 1912) mainly due to the presence of enlarged branchiae on chaetiger 5 (the first branchial chaetiger). This genus currently contains two deep-sea species, type species *B.sibogae* Horst, 1910 described from Malay Archipelago, depth of 1100 m and *B.balloniformis* Böggemann, 2009 described from the abyssal Atlantic. Similarly, *Chloenopsis* Fauchald, 1977 has been established to accommodate species originally described by McIntosh (885) as *Chloeneaatlantica* from the Canary Islands, depth ca. 2800 m. The validity of these genera and their separation from *Chloeia* has never been phylogenetically tested, but has been previously questioned (Böggemann 2009).

#### ﻿ Bathychloeia cf. balloniformis

Taxon classificationAnimaliaAmphinomidaAmphinomidae

Böggemann, 2009

064DDABA-5F87-57E8-85C3-5C0832677994

**Figure 2.**
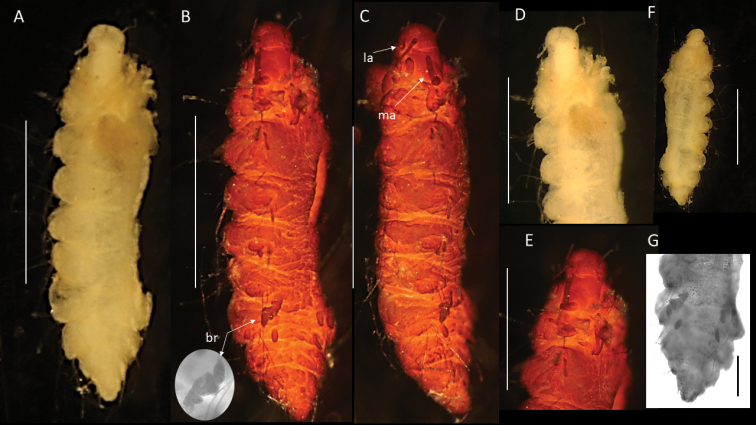
Bathychloeiacf.balloniformis (specimen, NHMUK ANEA.2022.630) **A** preserved specimen in dorsal view **B** specimen stained with Shirlastain in dorsal view, branchia (br) on chaetiger 6 marked by arrow, insert – detail of the same **C** specimen stained with Shirlastain in lateral view, median antenna (ma) and lateral antenna (la) marked by arrow **D** anterior end in dorsal view **E** anterior end in dorsal view stained with Shirlastain **F** preserved specimen in ventral view **G** posterior end in dorsal view stained with Shirlastain. Scale bars: 1 mm (**A-F**); 250 µm (**G**).

**Figure 3.**
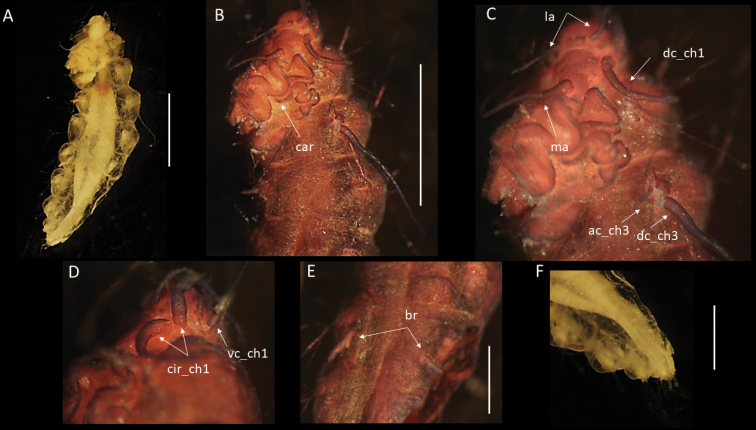
Bathychloeiacf.balloniformis (specimen NHMUK ANEA.2022.631) **A** preserved specimen in dorsal view **B** anterior end in dorsal view with caruncle (car) marked by arrow, specimen stained with Shirlastain **C** anterior end in dorsal view with median antenna (ma), lateral antennae (la), cirri (dc) on chaetiger 1 and on chaetiger 3 (ac, dc) marked by arrows, specimen stained with Shirlastain **D** anterior end in lateral view, cirri of chaetiger 1 marked by arrows, ventral cirrus (vc) **E** branchiae on chaetiger 6 (arrows) **F** posterior end in dorsal view with two anal cirri. Scale bars: 1 mm (**A, B**); 500 µm (**E, F**).

**Figure 4.**
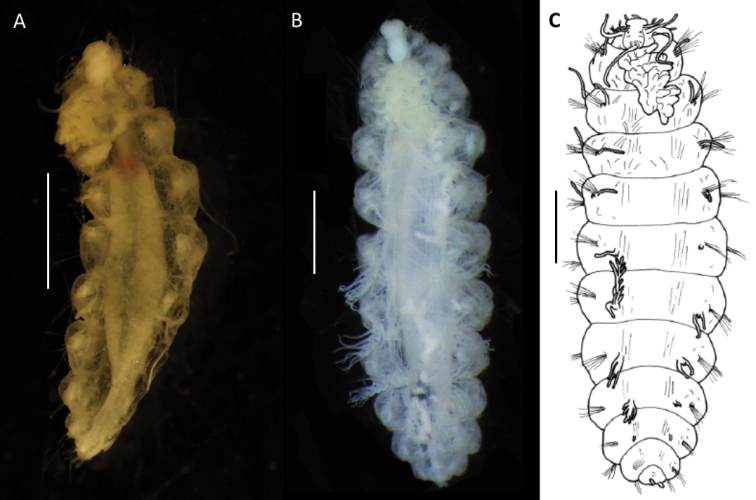
Comparative figure of **A**Bathychloeiacf.balloniformis (CCZ specimen NHMUK ANEA.2022.631) in dorsal view **B**Amphinomidae sp. (AM W.52607) specimen in dorsal view **C** drawing of *Bathychloeiaballoniformis* in dorsal view (after Böggemann 2009). All scale bars: 1 mm.

Figs 2A–G , 3A–F , 4A

##### Material examined.

NHM_2107, NHMUK ANEA 2022.630, coll. 20/03/2015, EBS, 19.46457, -120.02542, 4026 m, APEI-6, http://data.nhm.ac.uk/object/c79b4600-e8e9-4484-b06a-e18330a1421d; NHM_2109, NHMUK ANEA 2022.631, coll. 20/03/2015, EBS, 19.46457, -120.02542, 4026 m, APEI-6, http://data.nhm.ac.uk/object/ac3dd714-64ac-44ea-9168-22437dc3cfba.

##### Comparative material.

Amphinomidae spp.; AM. W.52607; 3 specimens; IN2017; sta. V03_110; 4005 m; South Pacific, Australia, off Fraser Island (-25.220, 154.160); col. 11/06/2017; EBS.

##### Diagnosis.

This very small species is represented by two specimens, up to 2.9 mm long and 0.75 mm wide for ten chaetigers. Body compact, spindle-shaped, of bloated appearance (Figs 2A–C, F, 3A, 4A). Preserved specimens pale yellow (Figs 2A, 3A), live specimens translucent to slightly tanned.

Prostomium rounded, longer than wide; anterior lobe broadly rounded, bearing a pair of cirriform lateral antennae (Figs 2C, 3C), a pair of slightly shorter ventrolateral palps and posteriorly prostomium with longer median antenna (Figs 2C, 3C). Prostomium with pair of very small reddish eyes (Fig. 2A, D); posteriorly extended into a conspicuous caruncle reaching the anterior margin of 3^rd^ chaetiger; caruncle large, ramified, and with deeply folded margins (Fig. 2B, C).

Parapodia biramous. Parapodial appendages often broken off, where attached dorsal, lateral and ventral cirri observed, including on chaetiger 1 (Fig. 3C, D). In anterior chaetigers cirri slightly more robust with thickened bases. Bipinnate branchiae observed only on chaetiger 6, with a large primary stalk and up to seven short lateral branches (Figs 2B, C, G, 3E). Branchiae on preceding segments likely absent (no scars or stalks observed), but those on subsequent segments likely present, but damaged (scars or stalks observed). Chaetae mostly broken off, only few long bifurcate noto- and neurochaetae arising directly from body wall observed, where observed prongs smooth. Pygidium as a conical lobe (Fig. 2G), with dorsal anus and with a pair of short terminal cirri (Fig. 3F).

##### Molecular information.

**Figure 5.**
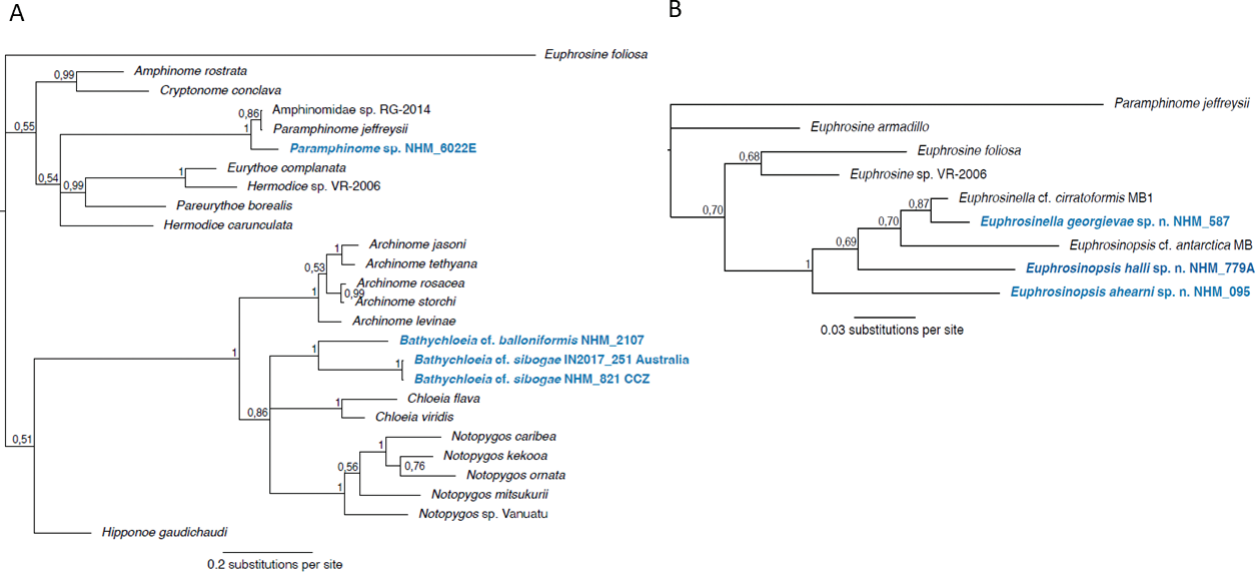
Majority-rule consensus trees from the Bayesian analyses with posterior probability values on nodes. Taxon names highlighted in blue are news species or new sequences for already known species. **A**Amphinomidae phylogenetic tree using a combined datasets for COI, 16S, and 18S with 26 terminal taxa of which *Euphrosinefoliosa* (Euphrosinidae) was used as a root **B**Euphrosinidae phylogenetic tree using a combined datasets for 16S and 18S with nine terminal taxa of which *Paramphinomejeffreysii* (Amphinomidae) was used as a root.

Specimen, NHMUK ANEA.2022.630, was successfully sequenced for 16S, 18S and COI while for specimen, NHMUK ANEA.2022.631, only 16S was obtained (Table 1). There were no identical sequences for either 16S or COI found on the GenBank. In the phylogenetic tree this species falls out as sister taxon to Bathychloeiacf.sibogae and the *Bathychloeia* clade is in an unresolved trichotomy with clades consisting of species from the genera *Chloeia* and *Notopygos*, although this trichotomy has low support (Fig. 5A).

##### Remarks.

The CCZ-collected specimens correspond morphologically to another abyssal species *Bathychloeiaballoniformis* Böggemann, 2009 described from Cape and Guinea Basins in SE Atlantic, 5048–5144 m depth. The specimens agree in small, spindle-shaped body, having ca. 10 chaetigers, the form of greatly folded and crenulated caruncle and the form and distribution of branchiae (see comparative Fig. 4A, C). Additionally, specimens recently collected from the abyssal South Pacific (ca. 4000 m) as part of the RV ‘Investigator’ voyage ‘Sampling the Abyss’ were made available for examination (see also Gunton et al. 2021). Originally identified as Amphinomidae sp. (Fig. 4B), morphologically these specimens also agree well with the description of *Bathychloeiaballoniformis* from the NE Atlantic (Fig. 4C) and with CCZ-collected specimens (Figs 2, 3, 4A). However, molecular work on specimens from South Pacific was not successful and no molecular work was carried out on specimens from the abyssal Atlantic (Böggemann pers. comm.). Due to lack of molecular data from the other locations, we cautiously ascribe CCZ-collected specimens to Bathychloeiacf.balloniformis.

##### Distribution.

Central Pacific Ocean, Eastern CCZ, in the Area of Particular Environmental Interest, ‘APEI-6’ only (Fig. 1).

#### ﻿ Bathychloeia cf. sibogae

Taxon classificationAnimaliaAmphinomidaAmphinomidae

Horst, 1910

61CF8FF7-DB0B-5FCA-BD0B-8469C57FB331

**Figure 6.**
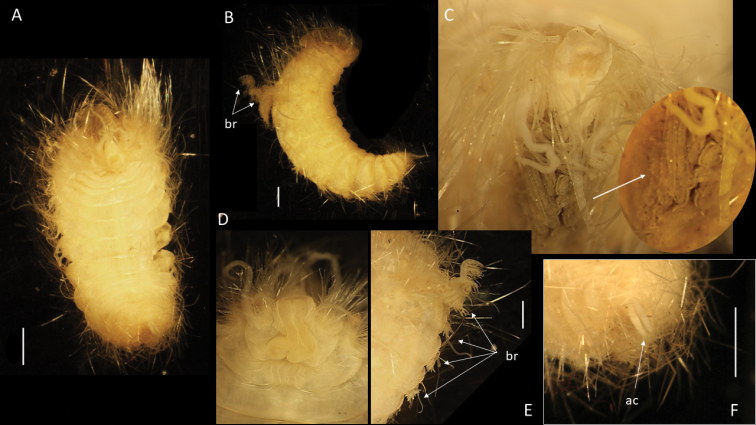
Bathychloeiacf.sibogae (specimen NHMUK ANEA.2022.633) **A** preserved specimen in ventral view **B** specimen in lateral view with large pair of branchiae on chaetiger 5 marked by arrows **C** anterior end in dorsal view with prostomium and caruncle (insert) **D** detail of mouth and anterior end in ventral view **E** branchiae on chaetiger 5-8 marked by arrows **F** detail of anterior end with anal cirri marked by arrow. Scale bars: 1 mm. Abbreviations: br – branchiae, ac – anal cirri.

**Figure 7.**
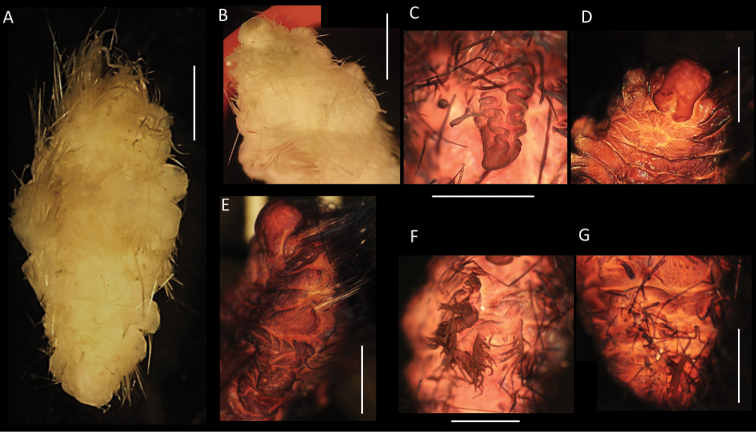
Bathychloeiacf.sibogae (specimen NHMUK ANEA.2022.634) **A** preserved specimen in dorsal view **B** anterior end in dorsal view **C** detail of the posterior end of caruncle **D** anterior end and mouth in ventral view **E** anterior end in lateral view with long ventral cirri on chaetiger 1 **F** large pair of branchiae on chaetiger 5 **G** posterior end in dorsal view with pair of anal cirri. Scale bars: 1 mm.

**Figure 8.**
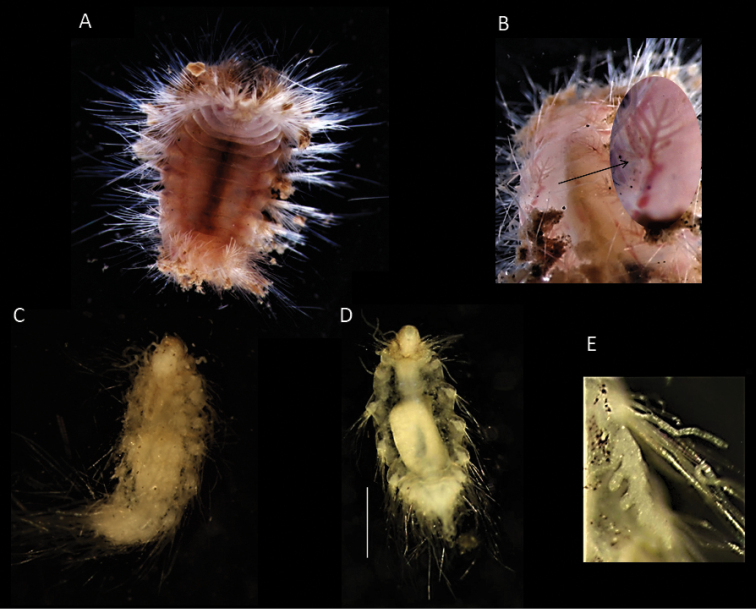
Bathychloeiacf.sibogaeCCZ-collected specimens **A** large live specimen (NHMUK ANEA.2022.633) in ventral view **B** live specimen (NHMUK ANEA.2022.633) with midbody segments and associated branchiae in dorsal view, insert – the detail of branchiae from chaetiger 9 **C** small (juvenile) preserved specimen (NHMUK ANEA.2022.635) in dorsolateral view **D** small (juvenile) preserved specimen (NHMUK ANEA.2022.632) in dorsal view **E** detail of enlarged branchiae on chaetiger from specimen (NHMUK ANEA.2022.632). Scale bar: 1 mm.

**Figure 9.**
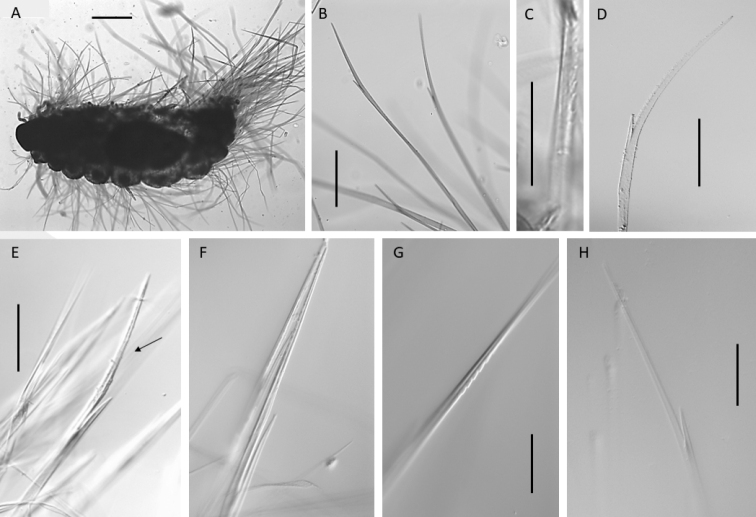
Bathychloeiacf.sibogae (specimen NHMUK ANEA.2022.635) **A** overview of specimen in dorsal view **B** slender furcate notochaetae **C** prong with distinct serration on inner margin **D** slender short furcate chaeta **E** long stout furcate chaeta (marked by arrow) **F** smooth prongs **G** faintly serrated prong, outer margin **H** slender furcate chaeta. Scale bars: 250 µm (**A**); 100 µm (**B**), 50 µm (**C, D, G, H**); 100 µm (**E**).

**Figure 10.**
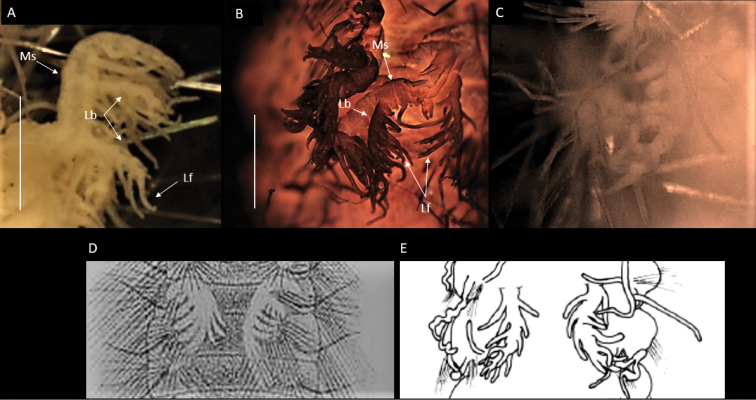
Comparative figure of form of enlarged branchiae from chaetiger 5 showing variation in development of long branchial filaments, Ms – Main stalk, Lb – lateral branches, Lf – long filaments **A**Bathychloeiacf.sibogaeCCZ specimen, NHMUK ANEA.2022.633, in lateral view **B***Bathychloeiacf.sibogae*CCZ specimen, NHMUK ANEA.2022.634, specimen stained with Shirlastain, in dorsal view **C**Bathychloeiacf.sibogae South Pacific specimen NHM_215 in dorsal view **D***Bathychloeiasibogae* after Horst (1912)**E***Bathychloeiasibogae* after Böggemann (2009). Scale bars: 1 mm.

Figs 6A–F , 7A–G , 8A–E , 9A–H , 10A, B

##### Material examined.

NHM_6880HW, NHMUK ANEA 2022.632, coll. 12/05/2021, box core, 10.3244, -117.1875, 4280 m, NORI-D, http://data.nhm.ac.uk/object/06f82805-e608-4715-af62-ab1d44df2a79; NHM_0821, NHMUK ANEA 2022.633, coll. 20/02/2015, EBS, 12.53717, -116.60417, 4425 m, UK-1, http://data.nhm.ac.uk/object/73a7200a-ae19-4c0c-8381-8d4509a318cf; NHM_2906, NHMUK ANEA 2022.634, coll. 20/02/2015, EBS, 12.53717, -116.60417, 4425 m, UK-1, http://data.nhm.ac.uk/object/d3848fcf-4cb2-49fd-b49c-e09422419a70; NHM_2115, NHMUK ANEA 2022.635, coll. 20/03/2015, EBS, 19.46457, -120.02542, 4026 m, UK-1, http://data.nhm.ac.uk/object/2cbc0d92-247c-4197-bd7a-4715adb5e8f4; NHM_3539, NHMUK ANEA 2022.636, coll. 02/03/2020, box core, 14.11729, -116.46109, 4148 m, OMS, http://data.nhm.ac.uk/object/083df63d-60e7-48ae-95c4-6a11a61b01e8; NHM_8922, NHMUK ANEA 2022.637, coll. 14/05/2018, box core, 10.39247, -117.46752, 4350 m, NORID-D, http://data.nhm.ac.uk/object/805f34aa-ec4f-4318-b18b-46447350aa1e.

##### Comparative material.

Bathychloeiacf.sibogae; NHMUK ANEA.2022.455-456; 2 specimens; IN_251; IN2017_V03_110; 4010 m; South Pacific, Australia, off Fraser Island (-25.220, 154.160); col. 11/06/2017; EBS. Bathychloeiacf.sibogae; AM W.52608 (1 specimen); IN2017_V03_103; South Pacific, Australia, off Moreton Bay (-27.008, 154.223); 4260 to 4280 m; coll. 10/06/2017; EBS. Bathychloeiacf.sibogae; AM W.52609 (2 specimens); IN2017_V03_096; South Pacific, Australia, off Byron Bay (-28.678, 154.204); 2591 to 2566 m; coll. 07/06/2017; EBS. Bathychloeiacf.sibogae; AM W.52610 (1 specimen); IN2017_V03_102; South Pacific, off Moreton Bay (-27.009, 154.223); 4274 to 4264 m; coll. 10/06/2017; beam trawl.

##### Diagnosis.

Body size variable, up to 18 mm long and 6 mm wide for larger specimens with 15 or 16 chaetigers (Figs 6A, 7A, 8A); smaller specimens up to 2 mm long and 0.7 mm wide (Fig. 8C, D). Body oval and compact; tapering anteriorly and posteriorly with mid-body chaetiger widest. Body pale yellow in alcohol, with rusty brown pigmentation in the mid furrow on anterior part of prostomium (Fig. 6C). Live large specimens pink in colour (Fig. 8A).

Prostomium indistinctly divided into an anterior and a posterior lobe; tightly surrounded by reduced first chaetigerous segment. Anterior lobe rounded, bearing a pair of lateral cirriform antennae plus a pair of slightly shorter ventrolateral palps. Posterior lobe bell-shaped, ca. as long as wide. One pair of tiny red eyes present (Fig. 7B) Prostomium posteriorly extended into a conspicuous caruncle, reaching anterior margin of chaetiger 4, mostly free from the body wall, wedge-shaped with greatly undulated lateral margins with ca. 10 folds in larger specimens (Figs 6C, 7A, C) and simple “tongue-like” structure in smaller specimens (Fig. 8C). Slender cirriform style of median antenna ca. ½ the length of caruncle.

Parapodia biramous with distinctly separated rami, bearing cirri that are easily detached. Dorsal and lateral cirri slender, filiform, and long, present in notopodia; dorsal cirrus inserted dorsolaterally to notochaetae, lateral cirrus, inserted medially behind notopodial chaetae. Ventral cirri also filiform and elongated (particularly in chaetiger 1, Fig. 7E), but on subsequent chaetigers shorter than dorsal or lateral cirri. First pair of branchiae always on chaetiger 5 where greatly enlarged (Figs 6B, E, 7F, 10A, B). In large specimens branchiae with a large primary stalk with up to six smaller branches, each with many long slender lateral filaments (Figs 7F, 10A, B); subsequent branchiae (if detected) much reduced in size (Fig. 6E), bipinnate with up to seven branches (Fig. 8B). In smaller specimens branchiae of chaetiger 5 also enlarged, but simpler, bipinnate, with a slender main stalk and up to seven pairs of lateral filaments (Fig. 8E).

Notopodia with chaetae much larger and usually thicker than those of neuropodia, almost forming a “cage” over dorsum, obscuring the branchiae in some specimens, but very fragile and easily lost in most specimens, best preserved in juvenile specimens (Fig. 9A). Both noto- and neurochaetae bifurcate of various lengths, and thickness of shafts and prongs (Fig. 9B–H). Long prongs mainly with smooth margin (Fig. 9B, D, E, F, H) or variably developed serrated margin on inner (Fig. 9C) or outer margin (Fig. 9G). Pygidium with dorsal anus and a pair of digitiform elongated cirri (Fig. 7G).

##### Variation.

Molecular analysis suggests that smaller and larger specimens that differ predominantly in the form of caruncle and form of branchiae as described above, represent the same species. Therefore, the size difference likely represents different developmental changes.

##### Molecular information.

Only one CCZ specimen of B.cf.sibogae, specimen NHMUK ANEA.2022.633, was sequenced for all three genes, 16S, 18S and COI (Table 1). Three other specimens were successfully sequenced for COI and five for 16S only (Table 1). In addition, 16S (GenBank accession numbers ON900090 and ON900091) and COI (GenBank accession numbers ON903195 and ON903196) sequences were obtained from two specimens in the comparative material (NHMUK ANEA.2022. 455-456) that were collected from the abyssal South Pacific (off Australia). The COI sequences from this species matched four sequences on GenBank with accession numbers KJ736482-KJ736485, all four from other areas within CCZ (Janssen et al. 2015). In the phylogenetic tree, the specimens from CCZ and Australia fall as a sister taxon to Bathychloeiacf.balloniformis (Fig. 5B). The *Bathychloeia* clade is in an unresolved trichotomy with clades consisting of species from the genera *Chloeia* and *Notopygos*, although the trichotomy has low support (Fig. 5A). Uncorrected ‘p’ from a COI alignment of 534 characters shows values among the nine B.cf.sibogae specimens ranging from 0.0 to 0.015, while the lowest value between B.cf.sibogae and its closest relative in our phylogenetic analysis, B.cf.balloniformis, is 0.18.

##### Remarks.

The enlarged branchiae of chaetiger 5 suggest close affiliation of CCZ specimens to *Bathychloeiasibogae* Horst, 1910 described from the Banda Sea, depth of 1100 m. Since its original description and subsequent re-description (Horst 1912), specimens assigned to *B.sibogae* or B.cf.sibogae have been reported from vastly different geographic and more importantly bathymetric areas such as the Tasman Sea and off Kenya (Kirkegaard 1995), Guinea Basin in SE Atlantic in depths of 5048–5144 m (Böggemann 2009) and South Pacific in depths of 2566 m and 4260 m (Gunton et al. 2021). Further, Böggemann (2009) suggested that syntypes (BMNH1885.12.1.11) of *Chloenopsisatlantica* (McIntosh) from the NE Atlantic (Canary Islands, ca. 2800 m depth) may in fact belong to *B.sibogae* due to presence of similar branchiae and two notopodial cirri.

Although the original definition of *B.sibogae* given by Horst (1910) was limited, a more detailed re-description was provided by Horst later (Horst 1912). The type specimen ZMA.V.POL.124 was on loan and therefore not available for examination at the time of writing (J Bleeker, pers. comm.). Based of re-description of Horst (1912)CCZ specimens differ mainly in the presence of red eyes and form of branchiae that are bi-pinnate but with many slender filaments developed on lateral branches (Fig. 10A, B), a character not reported by Horst (Fig. 10D). CCZ specimens also correspond well with those reported by Böggemann (2009) from the abyssal SE Atlantic in having similar body shape and body size, presence of tiny eyes, form and distribution of parapodial cirri, well developed highly crenulated and folded caruncle (in larger specimens), enlarged branchiae on chaetiger 5 and form of pygidial cirri. However, Böggemann (2009) did not report the presence of long filaments of branchial lateral branches (Fig. 10E). Additionally, specimens identified as B.cf.sibogae collected from the abyssal South Pacific were also available for morphological and molecular comparison (see also Gunton et al. 2021). Morphologically the South Pacific specimens agreed with those collected from CCZ, with long filaments on lateral branchial branches either present or absent (Fig. 10C). Significantly, the molecular data (CO1, 16S and 18S markers) suggested that CCZ and South Pacific specimens belong to the same species, therefore the presence/absence of filaments on lateral branchial branches may be a matter of preservation or developmental character. Currently, no molecular data are available from the SE Atlantic specimens or from the type locality. Although it is unlikely that abyssal specimens belong to the same species as that described by Horst (1910) from 1100 m, due to lack of molecular data from type locality we cautiously ascribe CCZ-collected specimens to Bathychloeiacf.sibogae.

##### Distribution.

Central Pacific Ocean, Eastern CCZ, in the exploration areas UK-1, OMS, NORI-D (Fig. 1) and based on previous study in GBR (German) and IFREMER (French) exploration areas (Janssen et al. 2015). Abyssal South Pacific, off Australia, ca. 4000 m.

##### Ecology.

It is of interest that a closely related form to the CCZ species known as *Cholenopsisatlantica* (McIntosh, 1885) has been described in association with a sponge growing on a dead coral coated with manganese of peroxide (McIntosh 1885), while the CCZ species has been collected from the sediment associated with manganese nodules.

#### ﻿ Paramphinome

Taxon classificationAnimaliaAmphinomidaAmphinomidae

M. Sars in G. Sars, 1872

C94FDE83-87B7-5AF4-9F16-046745D885DE

##### Type species.

*Paramphinomepulchella* M. Sars in G. Sars, 1872.

##### Diagnosis.

Small but long long-bodied forms. Prostomium posteriorly with Y-shaped or elongated caruncle. Branchiae comb-shaped, limited to the anterior chaetigers. First chaetiger with curved hooks in notopodia.

#### ﻿ Paramphinome

Taxon classificationAnimaliaAmphinomidaAmphinomidae

sp. NHM_6022E

0DD719E9-2EAA-57DD-9D76-B40B9D99A178

Figs 11A–C , 12A–I

##### Material examined.

NHM_1167D, NHMUK ANEA 2022.638, coll. 26/02/2015, EBS, 12.11550, -117.16450, 4100 m, OMS, http://data.nhm.ac.uk/object/fd4902df-aef2-44cf-991f-31905434c2a1; NHM_4044, NHMUK ANEA 2022.639, coll. 06/03/2020, box core, 13.27406, -116.69997, 4185 m, UK-1, http://data.nhm.ac.uk/object/56235559-3f2c-426e-b4cd-37462593a4ba; NHM_6022E, NHMUK ANEA 2022.640, coll. 13/11/2020, box core, 10.35780, -117.15931, 4284 m, NORI-D, http://data.nhm.ac.uk/object/bd4b405d-3e56-4671-909e-fdf9c3e7fbcf.

##### Diagnosis

**Figure 11.**
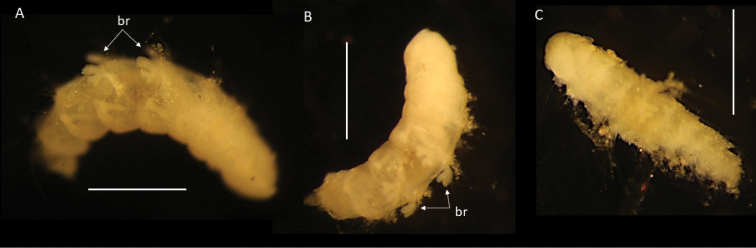
*Paramphinome* sp. NHM_6022E **A, B** preserved specimen NHMUK ANEA.2022.638 in dorsolateral view, branchiae (br) of chaetigers 4 and 5 marked by arrows **C** preserved specimen, NHMUK ANEA.2022.639, in dorsal view. Scale bars: 500 µm.

**Figure 12.**
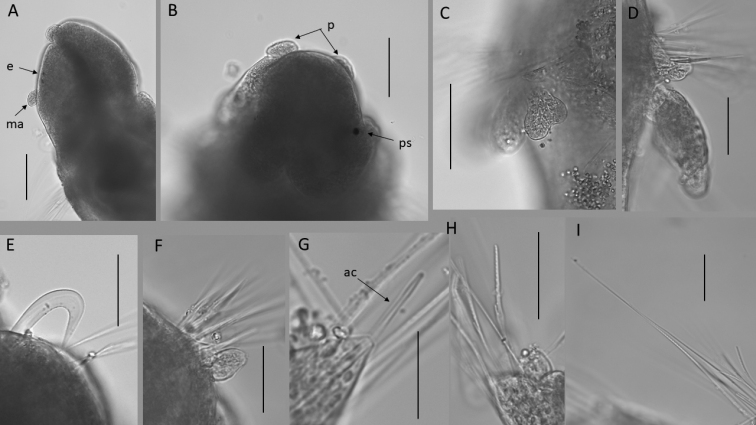
*Paramphinome* sp. NHM_6022E (specimen, NHMUK ANEA.2022.638) **A** anterior end in lateral view, with eyes and median antenna **B** prostomium in ventral view with palps and pigmented spots **C** detail of branchiae from chaetiger 4 in dorsolateral view **D** branchiae and dorsal lobe from chaetiger 4 in lateral view **E** notopodial hook from chaetiger 1 **F** dorsal lobe from chaetiger 2 **G** protruding acicular spine (ac) **H** spinose bifurcate chaeta **I** long spinose neurochaeta. Scale bars: 100 µm (**A-C**); 50 µm (**D–I**). Abbreviations: e – eyes, ma – median antenna, p – palps, ps – pigmented spots, ac – acicular spines.

(after Fauchald (1977)). All very small, poorly preserved and posteriorly incomplete specimens (Fig. 11A–C). Specimen NHMUK ANEA.2022.638, 1.65 mm long and 0.35 mm wide for ca. 7 discernible chaetigers. Prostomium broad, rounded, slightly longer than wide; with a pair of palps and lateral antennae and posteriorly with median antenna; all prostomial appendages tiny and globular to ovoid (Fig. 12A, B). Two pairs of tiny reddish eyes (Fig. 12A) in trapezoidal arrangement, plus a pair of tiny, pigmented spots present posteroventrally on prostomium (Fig. 12B). Caruncle as a low-lying lobe, reduced, difficult to observe.

Parapodia biramous. Dorsal cirri small and ovoid (Fig. 12F), ventral cirri not observed. Two pairs of branchiae present on chaetiger 4 and 5, comb-shaped with two main stalks branching into 4 terminal lobes (Figs 11A, B, 12C, D). Stout, distally strongly curved hook present in each notopodium of chaetiger 1 (Fig. 12E). Other observable chaetae include stout spines, slightly subdistally swollen (Fig. 12G); slender bifurcate chaetae, their prongs significantly differing in length, the long prong marginally serrated (Fig. 12H) and slender, long, smooth chaetae (Fig. 12I). Posterior segments and pygidium not observed.

##### Molecular information.

Only one specimen, NHMUK ANEA.2022.640, was successfully sequenced for 16S and 18S (Table 1). There were no identical sequences for 16S on GenBank. In the phylogenetic tree this species falls as a sister taxon to *Paramphinomejeffreysii* and an unidentified specimen, Amphinomidae sp. RG-2014 (Fig. 5A).

##### Remarks.

Three very small posteriorly incomplete specimens were collected in CCZ samples. They differ from known species by its very small size and low number of branchial pairs (only two pairs) and undeveloped prostomial appendages, which are tiny and globular. While body size, number of segments and number of branchial pairs were previously linked to developmental stages (e.g., Kudenov 1993; Barroso and Paiva 2008), we believe that the three specimens presented here, collected during three different cruises up to eight years apart, represent a small-bodied species rather than juveniles.

Of the known deep-sea *Paramphinome* species, none were described from the abyssal depths. *Paramphinomepacifica* Fauchald & Hancock, 1981 has been described from NE Pacific Ocean: off central Oregon (USA), 1800–2900 m; (type locality: Cascadia Abyssal Plain, 2860 m). *Paramphinomeaustralis* Monro, 1930 has type locality off Signy Island, South Orkney Islands, Southern Ocean in depths between 244–344 m, although it has been widely reported from the Southern Ocean (Kudenov 1993) and also the abyssal Atlantic (Böggemann 2009). *Paramphinomeposterobranchiata* Barroso & Paiva, 2008 has type locality in South Atlantic, off Brazil at 1600 m depth. Finally, *P.jeffreysii* has type locality in St. Lawrence estuary (shallow depths), but has been widely reported, even from great depths (e.g., Gunton et al. 2015) and specimens ascribed to this taxon likely represents different species (see Fig. 5A).

It is likely that the CCZ-collected specimens represent a new species; however, their tiny size and poor morphological preservation prevent its formal description, therefore the specimens are assigned to morphospecies only.

##### Distribution.

Central Pacific Ocean, Eastern CCZ, the exploration contract areas UK-1, OMS, and NORI-D (Fig. 1).

### Euphrosinidae Williams, 1852

#### ﻿ Euphrosinella

Taxon classificationAnimaliaAmphinomidaAmphinomidae

Detinova, 1985

55DC13A5-1D62-5AA0-8ABB-C1094124B7F1

##### Type species.

*Euphrosinecirratoformis* Averincev, 1972.

##### Diagnosis

(modified from Kudenov (1993)). Prostomium with five appendages, including median antenna, two lateral antennae and two palps. Eyes present or absent. Caruncle free from the body wall for most of its length. Ringent chaetae absent.

##### Remarks.

Genus *Euphrosinella* was established by Detinova (1985) to accommodate species originally described by Averincev (1972) as *Euphrosinecirratoformis*. She distinguished Euphrosinella from Euphrosine mainly on the bases of the presence of five (instead of three) prostomial appendages. Characters such as the extent of fusion of caruncle to body wall and the absence of ringent chaetae were also suggested by Detinova (1985) but questioned by Kudenov (1993). The genus currently contains only two valid species, both from the deep waters and/or Antarctic habitats. *Euphrosinellacirratoformis* is widely distributed in the Antarctic waters and was considered circumpolar (Kudenov 1993), although recent molecular data suggest the presence of at least two distinct species (Brasier et al. 2016). The second species, *Euphrosinellapaucibranchiata* (Hartman 1960) has been described from deep waters off California (Santa Cruz Basin, 1737 m depth) and has not been widely reported since.

#### ﻿ Euphrosinella georgievae sp. nov.

Taxon classificationAnimaliaAmphinomidaAmphinomidae

1123F843-F2AF-5AF9-8BF8-B16606C2A8A6

https://zoobank.org/EE13C699-0E67-4060-893C-0AB0BB5E0045

**Figure 13.**
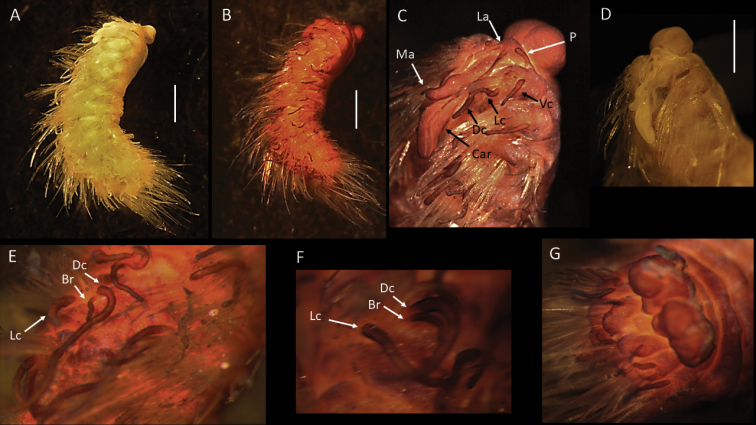
*Euphrosinellageorgievae* sp. nov. (holotype, NHMUK ANEA 2022.664) **A** preserved specimen in lateral views **B** specimen stained with Shirlastain in lateral view **C, D** detail of anterior end and prostomium with palps (P), lateral antennae (La), median antenna (Ma), caruncle (Car) and first chaetiger – dorsal cirrus (Dc), lateral cirrus (Lc) and ventral cirrus (Vc) marked by arrows **E, F** midbody chaetigers in dorsal view with branchiae (Br), dorsal (Dc) and lateral cirri (Lc) marked by arrows **G** pygidium in distal view. Scale bars: 1 mm. Abbreviations: Ma – median antenna, La – lateral antennae, P – palps, Vc – ventral cirrus, Lc – lateral cirrus, Dc – dorsal cirrus, Car – caruncle, Br – branchiae.

**Figure 14.**
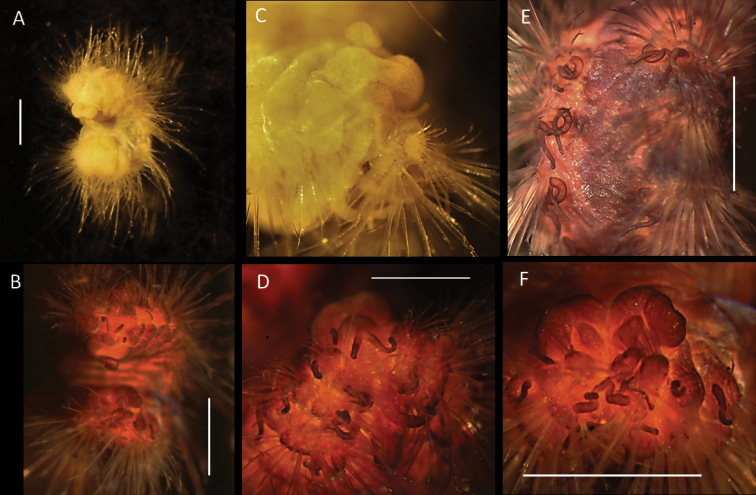
*Euphrosinellageorgievae* sp. nov. (paratype NHMUK ANEA.2022.662) **A** preserved specimen in dorsolateral view **B** specimen stained with Shirlastain, in dorsolateral view **C** detail of anterior end in dorsolateral view **D** detail of prostomium, with palps and lateral antennae **E** dorsal view of midbody chaetigers, showing branchiae and dorsal cirri **F** detail of pygidium in distal view. Scale bars: 1 mm.

**Figure 15.**
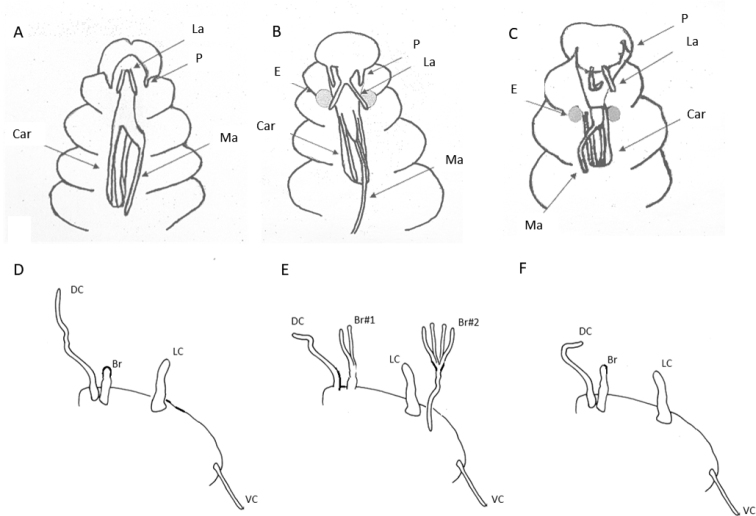
Diagrammatic representation of prostomial (**A–C**) and parapodial appendages from mid-body chaetigers (**D, E**) of CCZ-collected Euphrosinidae species (relative lengths preserved, but not drawn to scale) **A***Euphrosinellageorgievae* sp. nov. **B***Euphrosinopsisahearni* sp. nov. **C***Euphrosinopsishalli* sp. nov. **D***Euphrosinellageorgievae* sp. nov. **E***Euphrosinopsisahearni* sp. nov. **F***Euphrosinopsishalli* sp. nov. Abbreviations: P – palps, La – lateral antennae, Ma – median antenna, Car – caruncle, E – eyes, DC – dorsal cirrus, LC – lateral cirrus, VC – ventral cirrus, Br – branchiae.

**Figure 16.**
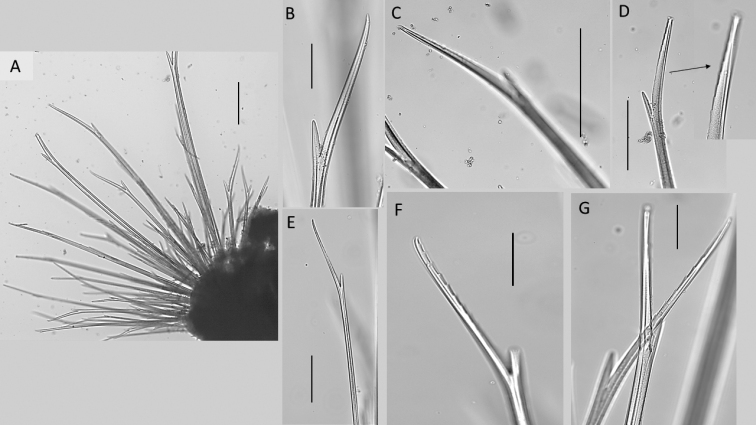
*Euphrosinellageorgievae* sp. nov. (specimen NHMUK ANEA.2022.658) **A** mid body parapodium **B** notochaeta from anterior tier **C** notochaeta from mid-tier **D** notochaeta from posterior tier, detail of serration (insert) **E-G** examples of neurochaetae. Scale bars: 100 µm (**A, B, E**); 200 µm (**C**); 250 µm (**D**); 50 µm (**F, G**).

Figs 13A–G , 14A–F , 15A , 16A–G

##### Material examined.

NHM_0587, NHMUK ANEA 2022.658, coll. 17/02/2015, EBS, 12.38624, -116.54867, 4202 m, UK-1, http://data.nhm.ac.uk/object/b7a0bf33-0dc4-4f61-90de-35865647a99f; NHM_0777, NHMUK ANEA 2022.659, coll. 20/02/2015, EBS, 12.38624, -116.54867, 4202 m, UK-1, http://data.nhm.ac.uk/object/a8f0e776-d7b6-4ec6-a549-78f40f17d89b; NHM_1737B, NHMUK ANEA 2022.660, coll. 11/03/2015, EBS, 12.17383, -117.19283, 4045 m, OMS, http://data.nhm.ac.uk/object/2784df45-eec0-4151-b12d-11d955985faa; NHM_0910, NHMUK ANEA 2022.661, coll. 23/02/2015, EBS, 12.57133, -116.6105, 4198 m, UK-1, http://data.nhm.ac.uk/object/05dfb32c-fc3a-4028-bf09-3eb840175661; NHM_1134 (paratype), NHMUK ANEA 2022.662, coll. 26/02/2015, EBS, 12.1155, -117.1645, 4100 m, OMS, http://data.nhm.ac.uk/object/00590d2b-f952-4c69-8bc2-ac2a408da17a; NHM_1514, NHMUK ANEA 2022.663, coll. 05/03/2015, EBS, 12.51316667, -116.491333, 4252 m, UK-1, http://data.nhm.ac.uk/object/96cb7b69-c0ea-4559-9b57-3abe6af4a4c7; NHM_2391 (holotype), NHMUK ANEA 2022.664, coll. 20/02/2015, EBS, 12.53717, -116.60417, 4425 m, UK-1, http://data.nhm.ac.uk/object/1ce8325f-74de-47de-a776-2dc50b8d69ae; NHM_4975, NHMUK ANEA 2022.665, coll. 28/10/2020, box core, 11.013923, -116.258737, 4234 m, NORI-D, http://data.nhm.ac.uk/object/677b7d67-d9cc-4ebd-8d79-cf5da5dc40da; NHM_6087, NHMUK ANEA 2022.666, coll. 14/11/2020, box core, 10.647709, -117.226887, 4183 m, NORI-D, http://data.nhm.ac.uk/object/eebfaecd-5ee2-49d6-be73-51eb91678487; NHM_5802, NHMUK ANEA 2022.667, coll. 11/10/2020, box core, 10.475094, -117.384872, 4306 m, NORI-D, http://data.nhm.ac.uk/object/c0e408e3-91e7-408f-aaef-3be86507105a; NHM_5057, NHMUK ANEA 2022.668, coll. 30/10/2020, box core, 10.929036, -116.26351, 4262 m, NORI-D, http://data.nhm.ac.uk/object/d92b1574-eccb-443c-a15d-b79357360b59; NHM_7235, NHMUK ANEA 2022.669, coll. 14/05/2021, box core, 10.3773, -117.1558, 4302 m, NORI-D, http://data.nhm.ac.uk/object/55637dc0-f9b9-4586-9bfb-7a821c785279; NHM_2908, NHMUK ANEA 2022.670, coll. 20/02/2015, EBS, 12.53717, -116.60417, 4425 m, UK-1, http://data.nhm.ac.uk/object/fba3fab7-ae4b-4415-a73c-a2ba6cd44601.

##### Diagnosis.

***Holotype*** (NHMUK ANEA.2022.664) complete (except for tissue sampled for DNA), 4.2 mm long and 1.1 mm wide without chaetae for 15 chaetigers (Fig. 13A). ***Paratype*** (NHMUK ANEA.2022.662) complete (except for tissue sampled for DNA), with 13 chaetigers (Fig. 14A). Body short, oval, flattened, pale yellow in alcohol (Figs 13A, 14A). Prostomium longer than wide, with five prostomial appendages (Fig. 15A). Pair of short slender palps (Figs 13C, 14C, D); pair of slender lateral antenna (Figs 13C, 14C, D); median antenna of caruncle with long thick ceratophore and slender cirrus only slightly longer than caruncle (Fig. 13C). Caruncle as oval lobe reaching to anterior margin of chaetiger 4, mostly free of body wall, with median keel and two pairs of lateral ridges, with median keel slightly thicker than the lateral ones (Fig. 13C, D). Eyes not observed.

Parapodia biramous, two rami well separated. Parapodia of chaetiger 1 well developed, not reduced, with dorsal, lateral, and ventral cirri (Fig. 13C). Parapodial appendages of subsequent chaetigers in the following dorsoventral order: dorsal cirrus, branchia, lateral cirrus, ventral cirrus (Fig. 15C). All cirri as single filaments of various length and thickness with dorsal cirrus longest (extending over three chaetigers in midbody) (Figs 13E, 14E); lateral cirrus shorter and more stout inserted in the middle of notochaetal bundle (Fig. 13E, F); ventral cirrus slightly shorter than lateral cirrus, slender. Branchia one per chaetiger, simple (unbranched) cirrus, inserted laterally to dorsal cirrus, very short (ca. ½ the length of mid body chaetiger) (Figs 13E, F, 14E, 15C).

All chaetae well developed, but prone to breakage, all bifurcate (Fig. 16A). Notochaetae in approximately three tiers; differing mainly in their length and thickness with notochaetae of mid tear longest and thickest (Fig. 16B–E); prongs mostly smooth (Fig. 16B, C, E) or few with very faint serration (Fig. 16D); ratio of short to long prong in the short chaetae of anterior tier ranges from 1:3.5-4 (where possible to establish); in long chaetae of mid-tier ratio ranges from 1:4 to 1:5 (where possible to establish). Ringent notochaetae absent. Neurochaetae less numerous and thinner than notochaetae; all bifurcate, of varying lengths, prongs with noticeable serration (Fig. 16F, G), few prongs appearing smooth. Pygidium with paired anal cirri, resembling cylindrical tube feet (Figs 13G, 14B, F).

##### Molecular information.

One specimen, NHMUK ANEA.2022.658, was sequenced for 16S and 18S genes, while the 13 additional specimens were sequenced for 16S only (Table 1). There were no identical sequences for 16S on GenBank. In the phylogenetic tree this species falls as a sister taxon to Euphrosinellacf.cirratoformis from Antarctica (Fig. 5B).

##### Remarks.

*Euphrosinellageorgievae* sp. nov. is consistent with the genus *Euphrosinella* in having five prostomial appendages, caruncle mostly free from body wall and absence of ringent chaetae. Only two valid species in *Euphrosinella* are currently known as mentioned earlier. A known Pacific species *Euphrosinellapaucibranchiata* can be distinguished by having some branchiae branched, as well as much shallower depth distribution of 1737 m in Santa Cruz Basin. *Euphrosinellageorgievae* sp. nov. is more similar to the Antarctic species *E.cirratoformis* in having simple unbranched branchiae. The species also share a similar form and length of caruncle and median antenna. However, the two species differ in the following characters: 1. The presence of two pairs of eyes in the Antarctic species, while CCZ specimens are eyeless; 2. Notochaetae arranged in 3 tiers in new species, rather than 2 tiers in the known species and 3. Branchiae are not developed on first chaetiger in *E.georgievae* sp. nov., whilst they are present in *E.cirratoformis*. As further evidence, the molecular data suggest that Antarctic specimens identified in a previous study as Euphrosinellacf.cirratoformis (see Brasier et al. 2016) are different to the specimens of *E.georgievae* sp. nov. (Fig. 5B).

##### Distribution.

Central Pacific Ocean, Eastern CCZ, the exploration areas UK-1, OMS, and NORI-D (Fig. 1).

##### Etymology.

This species is named for Dr. Magdalena Georgieva, who took part in ABYSSLINE expeditions to CCZ. She also collected Bathychloeiacf.sibogae specimens from CCZ used in this study as well as samples from the South Pacific during the RV Investigator cruise used here as a comparative material.

#### ﻿ Euphrosinopsis

Taxon classificationAnimaliaAmphinomidaEuphrosinidae

Kudenov, 1993

A9562A24-D8B2-535E-B6CC-56A931B18C04

##### Type species.

*Euphrosinopsisantipoda* Kudenov, 1993.

##### Diagnosis

(after Kudenov (1993)). Prostomium with five appendages, including median antenna, two lateral antennae, and two palps. No prostomial eyes, with one pair of eyes deeply embedded lateral to median antenna. Caruncle free from the body wall for most of its length.

##### Remarks.

The genus *Euphrosinopsis* is currently endemic to Antarctica and has been established to accommodate three known Antarctic species (Kudenov 1993): *E.antarctica* (Hartmann-Schröder & Rosenfeldt, 1992), *E.crassiseta* Kudenov, 1993, and *E.horsti* Kudenov, 1993. It is similar to *Euphrosinella* in having five prostomial appendages and reduced fusion of caruncle to the body wall. The main difference from both *Euphrosine* and *Euphrosinella* considered by Kudenov (1993) was the lack of prostomial eyes and presence of large, deeply embedded eyes positioned laterally to median antenna.

#### ﻿ Euphrosinopsis ahearni sp. nov.

Taxon classificationAnimaliaAmphinomidaEuphrosinidae

2EB737B3-F063-5C23-BC0C-BB27B39BD99A

https://zoobank.org/CF28C891-3176-4233-9FA4-34DB9451B395

**Figure 17.**
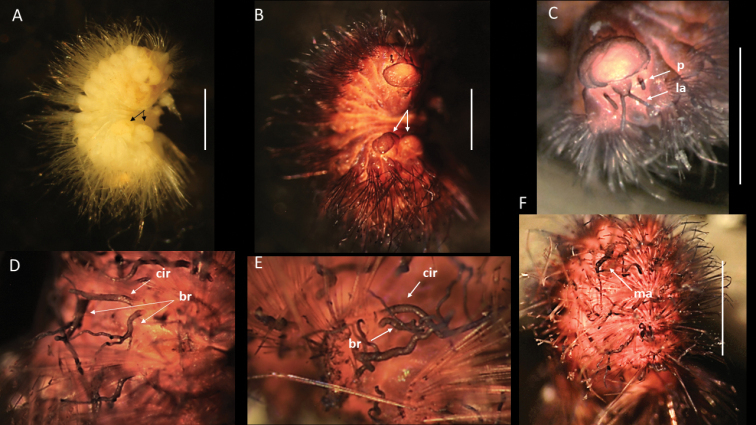
*Euphrosinopsisahearni* sp. nov. (holotype NHMUK ANEA.2022.652) **A** preserved specimen in dorsolateral view, pygidial cirri marked by arrows **B** specimen Shirlastained in dorsolateral view, pygidial cirri marked by arrows **C** detail of prostomium in dorsal view with palps (p) and lateral antennae (la) marked by arrows **D, E** branched branchiae (br) and lateral cirrus (cir) of midbody chaetigers **F** detail of midbody chaetigers, median antenna (ma) marked by arrow. Scale bars: 1 mm. Abbreviations: P – palps, la – lateral antennae, cir – cirrus, br – branchiae, ma – median antenna.

**Figure 18.**
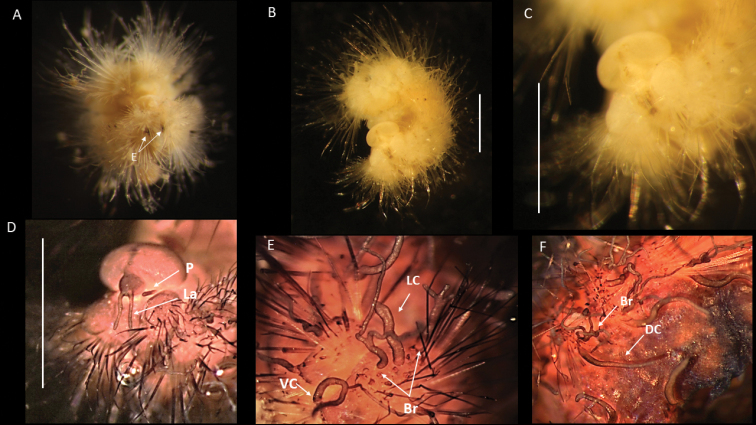
*Euphrosinopsisahearni* sp. nov. (paratype NHMUK ANEA.2022.654, unless stated otherwise) **A** live image of paratype (NHMUK ANEA.2022.646), with eyespots (E) marked by arrows **B** preserved specimen in dorsolateral view **C** anterior end in dorsolateral view **D** detail of prostomium (specimen stained with Shirlastain) with palp (P) and lateral antennae (La) marked by arrows **E** two pairs of branched branchiae (Br), lateral cirrus (LC) and ventral cirrus (VC) of midbody chaetigers **F** dorsal cirrus (DC) and branchia (Br) of midbody chaetigers. Scale bars: 1 mm. Abbreviations: E – eyes, P – palps, La – lateral antennae, Br – branchiae, LC – lateral cirrus, VC – ventral cirrus, DC dorsal cirrus.

**Figure 19.**
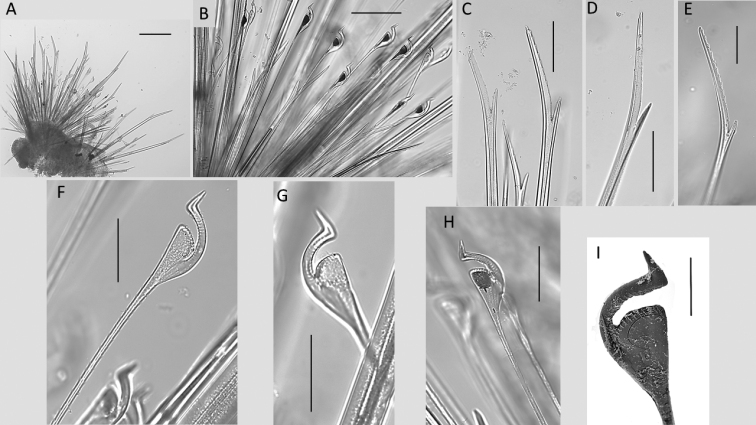
*Euphrosinopsisahearni* sp. nov. (paratype NHMUK ANEA.2022.646) **A** mid-body parapodium **B** notochaetae – anterior tier bifurcate and mid-tier ringent chaetae **C** bifurcate notochaetae posterior tier **D, E** details of variously serrated bifurcate notochaetae **F, G** examples of non-pigmented ringent notochaetae **H** details of pigmented ringent chaeta **I**SEM micrograph of ringent chaeta. Scale bars: 250 µm (**A**); 100 µm **(B–D**); 50 µm **(E–H**); 20 µm (**I**).

Figs 15B , 17A–F , 18A–F , 19A–I

##### Material examined.

NHM_0095, NHMUK ANEA 2022.644, coll. 11/10/2013, box core, 13.79335, -116.70308, 4081 m, UK-1, http://data.nhm.ac.uk/object/a351cb41-736c-4390-8ad8-02c0358b73e0; NHM_0888, NHMUK ANEA 2022.645, coll. 23/02/2015, EBS, 12.571333, -116.6105, 4198 m, UK-1, http://data.nhm.ac.uk/object/4d76b4e2-569d-4a17-9276-3ce721cbdf72; NHM_0551 (paratype, SEM), NHMUK ANEA 2022.646, coll. 17/02/2015, EBS, 12.386243, -116.54867, 4202 m, UK-1, http://data.nhm.ac.uk/object/241b828d-a574-47f2-995d-0bdef239c427; NHM_5042, NHMUK ANEA 2022.647, coll. 30/10/2020, box core, 10.92936, -116.26351, 4262 m, NORI-D, http://data.nhm.ac.uk/object/1662fd8b-54a5-4f97-9083-02dbb2df7e39; NHM_1737A, NHMUK ANEA 2022.648, coll. 11/03/2015, EBS, 12.17383, -117.19283, 4045 m, OMS, http://data.nhm.ac.uk/object/4f372c07-c466-4b6c-91a9-229cd7c7a17d; NHM_1876, NHMUK ANEA 2022.649, coll. 13/03/2015, EBS, 12.0415, -117.21717, 4094 m, OMS, http://data.nhm.ac.uk/object/6ad5c2b3-ece8-4195-a19f-3913de511e71; NHM_0550, NHMUK ANEA 2022.650, 17/02/2015, EBS, 12.386243, -116.54867, 4202 m, UK-1, http://data.nhm.ac.uk/object/92791783-35c2-4fbf-80b0-2b074ef70828; NHM_1302, NHMUK ANEA 2022.651, coll. 01/03/2015, EBS, 12.257333, -117.3021667, 4302 m, OMS, http://data.nhm.ac.uk/object/7aabe644-2ec6-4671-8c1a-f826eeeb0b46; NHM_1302A (holotype), NHMUK ANEA 2022.652, coll. 01/03/2015, EBS, 12.257333, -117.3021667, 4302 m, OMS, http://data.nhm.ac.uk/object/479933d3-9943-4d87-a1b8-ea120bd8f4ee; NHM_1737, NHMUK ANEA 2022.653, coll. 11/03/2015, EBS, 12.17383, -117.19283, 4045 m, OMS, http://data.nhm.ac.uk/object/2ca3e584-a68d-4ea5-98d2-75ce10515386;

NHM_1737C (paratype), NHMUK ANEA 2022.654, coll. 11/03/2015, EBS, 12.17383, -117.19283, 4045 m, OMS, http://data.nhm.ac.uk/object/efe95a8c-fc88-4849-ad26-1df3d292ef20; NHM_0616, NHMUK ANEA 2022.655, coll. 17/02/2015, EBS, 12.386243, -116.54867, 4202 m, UK-1, http://data.nhm.ac.uk/object/4758bf19-c6d0-42e0-b5ba-e83e203d2e18; NHM_0759, NHMUK ANEA 2022.656, coll. 20/02/2015, EBS, 12.53717, -116.60417, 4425 m, UK-1, http://data.nhm.ac.uk/object/b0f9162f-a861-4eb2-89a1-ce25c2bd09c4; NHM_1839, NHMUK ANEA 2022.657, coll. 12/03/2015, box core, 12.0999, -117.1966, 4051 m, OMS, http://data.nhm.ac.uk/object/02a5ace7-841e-4f50-bf03-57ba21f02f7c.

##### Diagnosis.

***Holotype*** (NHMUK ANEA.2022.652) complete (except for tissue sampled for DNA), 2.2 mm long and 0.8 mm wide without chaetae for 12 chaetigers (Fig. 17A–F). ***Paratype*** (NHMUK ANEA.2022.654) complete (except for tissue sampled for DNA), 2.8 mm long and 1 mm wide for 12 chaetigers (Fig. 18A–F). ***Paratype*** (NHMUK ANEA.2022.646, SEM specimen) anterior fragment only (Fig. 19A–I). Body short, oval, flattened, pale yellow in alcohol (Figs 17A, 18B), with a patch of light brown pigmentation on prostomium (Fig. 18C). Live specimen pale with blueish hues (Fig. 18A). Prostomium longer than wide, with 5 prostomial appendages (Fig. 15B). Pair of short slender palps (Figs 17C, 18D); pair of slender lateral antenna (at least twice the length of palps) (Figs 17C, 18D) and median antenna of caruncle with long thick ceratophore and very long slender cirrus reaching dorsally to chaetiger 7 (Fig. 17F). Caruncle as oval lobe reaching to anterior margin of chaetiger 4, mostly free of body wall, with median keel and two pairs of lateral ridges, median keel slightly thicker than the lateral ones. Single pair of large, spherical eyes, deeply embedded, lateral to median antenna and caruncle (Figs 15B, 18A).

Parapodia biramous, two rami well separated. Parapodia of chaetiger 1 well developed, not reduced, parapodial cirri, branchiae or ringent chaetae not observed. In subsequent parapodia, parapodial appendages in the following dorsoventral order: dorsal cirrus, 1^st^ branchia, lateral cirrus, 2^nd^ branchia, ventral cirrus (Fig. 15E). Dorsal cirrus as a single very long filament (extending over two chaetigers in mid-body segments) (Fig. 18F); the lateral cirrus as a shorter, stouter filament (Fig. 17D, E); ventral cirri often missing, when observed, very slender (Fig. 18E). Branchiae up to two pairs present per segment in mid-body, first branchia attached laterally to dorsal cirrus (Figs 15E, 18F), second branchia attached laterally to lateral cirrus (Figs 15E, 17D), both branchiae branched with 2–4 very long and slender branches (Figs 15E, 17D, E, 18E, F).

Chaetae fragile, prone to breakage, of two main types: 1. Numerous, bifurcate chaetae arranged in three rows in notopodia; their shafts of various length and thickness (Fig. 19A); their prongs variable in length with short furcate chaetae in anterior tier having the ratio of short to long prong ca. 1:3.5 (where possible to establish), the prong ratio of longest chaetae in the mid tear 1:4–5 (where possible to establish); prongs mainly smooth or with faint serration (Fig. 19C–E); 2. Ringent chaetae (sensu Kudenov 1993) present in notopodia only, numerous (ca. 20 per notopodium) (Fig. 19A, B), composed of two curved prongs of unequal length and thickness, both with distinct serration, the long prong distally with slender tip, short prong broad and distally rounded (Fig. 19F–I). Neurochaetae numerous, but thinner than notochaetae, all bifurcate, of varying lengths prongs appearing smooth. Pygidium with paired anal cirri, resembling cylindrical tube feet (Fig. 17A, B).

##### Molecular Information.

Specimen (NHMUK ANEA.2022.644) was sequenced for 16S and 18S while 14 other specimens were sequenced for 16S only. There were no identical sequences for 16S on GenBank (Table 1). The relationships between *Euphrosinella* and *Euphrosinopsis* in the phylogenetic tree is unresolved (Fig. 5B). As COI sequencing was not successful in this study, all euphrosinid species are represented by only 16S and 18S.

##### Remarks.

The CCZ species agrees well with the genus *Euphrosinopsis* in having five prostomial appendages, caruncle partially free from the body wall and the presence of large, deeply embedded eyes lateral to median antenna and caruncle. However, this species shows differences from all known species in this genus, suggesting it belongs to a new species. *Euphrosinopsiscrassiseta* (type locality: Weddell Sea, 3697 m) can be easily distinguished by having only small, cirriform branchia per segment rather than two pairs of branched branchiae, by the absence of ringent chaetae and presence of coarsely serrated neurochaetae. *Euphrosinopsishorsti* (type locality: Pacific Antarctic Ridge, 3219–3255 m) also has only one very small, cirriform branchia per segment. Ringent chaetae are present in the known species, but they possess a distal tooth in the gap, which is absent in the new species. Finally, the most similar species, *Euphrosinopsisantarctica* can be distinguished by having up to three branchiae per segment, the first branched, but the others cirriform and style of median antenna of similar length to caruncle, rather than much longer as in the new species.

Thus, *Euphrosinopsisahearni* sp. nov. can be distinguished mainly by having two pairs of branched branchiae in midbody chaetigers, both with very long thin branches. That is also the main distinguishing character from its congener from the CCZ, *E.halli* sp. nov. also described in this study, which possess only single cirriform branchia in each parapodium. Both new species possess ringent notochaetae, that can be distinguished as follow: 1. They are numerous (ca. 20 per notopodium) and easily observed in *E.ahearni* sp. nov., whilst only few (ca. 5 per notopodium) can be found in *E.halli* sp. nov.; 2. The serration of inner margin is more pronounced in *E.ahearni* sp. nov. and 3. The distal tip is shorter and stubbier in *E.ahearni* sp. nov. Further, the caruncle is more developed in *E.ahearni* sp. nov. reaching to chaetiger four, not two as in *E.halli* sp. nov., and style of median antenna is much longer than caruncle in the former species, whilst they are ca. the same length in the latter.

##### Distribution.

Central Pacific Ocean, Eastern CCZ, the exploration areas UK-1, OMS, and NORI-D (Fig. 1).

##### Etymology.

This species is named for Patrick A’Hearn, technician from the University of Washington onboard the RV Thomas G Thompson.

#### ﻿ Euphrosinopsis halli sp. nov.

Taxon classificationAnimaliaAmphinomidaEuphrosinidae

B0F2724C-FF6E-503B-A683-A437C524C606

https://zoobank.org/83A9C528-4FBE-4836-82DC-C31BDA5C2B09

**Figure 20.**
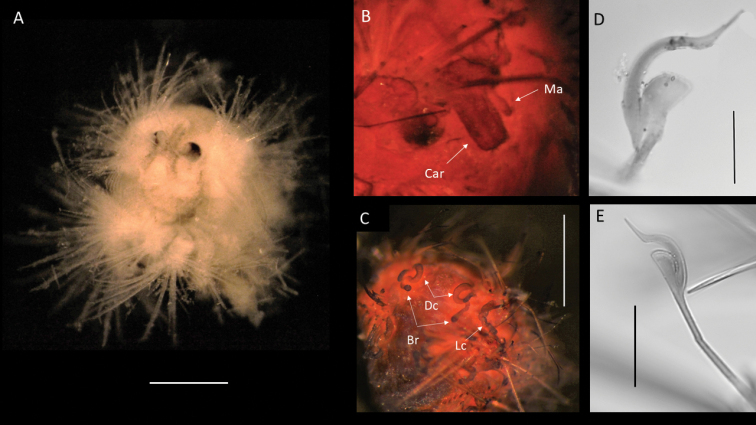
*Euphrosinopsishalli* sp. nov. **A** preserved holotype NHMUK ANEA.2022.642, eye spots visible **B** holotype NHMUK ANEA.2022.642 stained with Shirlastain, showing caruncle (car) and median antenna (Ma) **C** specimen NHMUK ANEA.2022.641 stained with Shirlastain, showing branchiae (Br) and dorsal cirri (Dc) and lateral cirrus (Lc) **D, E** ringent chaetae from specimen NHMUK ANEA.2022.641. Scale bars: 500 µm (**A, C**); 25 µm (**D**); 50 µm (**E**). Abbreviations: Ma – median antenna, Car – caruncle, Br – branchiae, Dc – dorsal cirrus, Lc – lateral cirrus.

**Figure 21.**
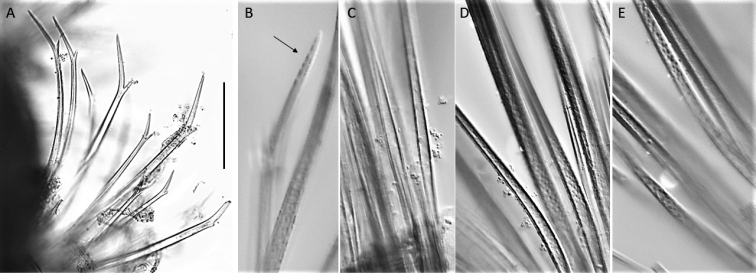
*Euphrosinopsishalli* sp. nov. (holotype NHMUK ANEA.2022.642) **A** long and short furcate notochaetae **B** faint serration of long prong (marked by arrow) **C** smooth shafts of dorsalmost notochaetae **D** shafts with file-like teeth of middle chaetae **E** shafts with file-like teeth of ventralmost chaetae. Scale bar: 250 µm.

Figs 15C , 20A–E , 21 A–E

##### Material examined.

NHM_0779, NHMUK ANEA 2022.641, coll. 20/02/2015, EBS, 12.53717, -116.60417, 4425 m, UK-1, http://data.nhm.ac.uk/object/1a683870-d904-4c2c-bf1a-a34ead0a42fc; NHM_4339 (holotype), NHMUK ANEA 2022.642, coll. 11/03/2020, box core, 12.17997, -117.065277, 4117 m, UK-1, http://data.nhm.ac.uk/object/670dfd34-338d-4edc-8856-b0a9a728efc9; NHM_6018 (paratype), NHMUK ANEA 2022.643; coll. 13/11/2020, box core, 10.35780, -117.15931, 4284 m, NORI-D, http://data.nhm.ac.uk/object/ab26e2ea-ab87-4013-8106-e817c0485cc9.

##### Diagnosis.

***Holotype*** (NHMUK ANEA.2022.642) complete (except for tissue sampled for DNA), 1.3 mm long and 0.75 mm wide without chaetae for 11 chaetigers. ***Paratype*** (NHMUK ANEA.2022.643) complete (except for tissue sampled for DNA), 1.5 mm long and 0.75 mm wide for 12 chaetigers. Body short, oval, flattened, pale yellow in alcohol (Fig. 20A). Prostomium longer than wide, with 5 prostomial appendages (Fig. 15C). Pair of short slender palps; pair of slender lateral antenna; median antenna of caruncle with long thick ceratophore and slender cirrus of similar length to caruncle (Figs 15C, 20B). Caruncle as oval lobe reaching to chaetiger 2, mostly free of body wall, with median keel and two pairs of lateral ridges, median keel slightly thicker than the lateral ones (Fig. 20B). Single pair of large, spherical eyes, deeply embedded, lateral to median antenna and caruncle (Figs 15C, 20A).

Parapodia biramous, two rami well separated. With parapodial appendages observed dorso-ventrally as follow (Figs 15F, 20C): long slender dorsal cirrus, often curved into S-shape in middle chaetigers; single cirriform branchia attached laterally to dorsal cirrus; lateral cirrus similar to branchia in form, but more robust; slender cirriform ventral cirrus.

Chaetae fragile, prone to breakage, of two main types: 1. Numerous, bifurcate chaetae arranged in approximately three rows in notopodia; their shafts of various length and thickness (Fig. 21A); development of filelike teeth on shafts ranging from smooth (Fig. 21C) to well developed (Fig. 21D, E); their prongs variable in length with short furcate chaetae in anterior tier having the ratio of short to long prong ranging from 1:2-2.5 (where possible to establish), the prong ratio of longest chaetae in the mid tear ranging from 1:3.5-4 (where possible to establish); prongs mainly smooth or with extremely faint serration only visible under high magnification (Fig. 21B). 2. Ringent chaetae (sensu Kudenov 1993) present in notopodia only, few in numbers (ca. 5 per notopodium), composed of two curved prongs of unequal length and thickness, both with indistinct serration, the long prong distally with slender elongated tip, short prong broad and distally rounded (Fig. 20D, E). Neurochaetae similar less numerous, thinner, and shorter than notochaetae, all bifurcate, prongs appearing smooth, often broken off. Pygidium with pair of cirri resembling cylindrical tube feet.

##### Molecular information.

Specimen NHMUK ANEA.2022.641, was sequenced for 16S and 18S while paratype NHMUK ANEA.2022.643 and holotype NHMUK ANEA.2022.642 were sequenced for 16S only (Table 1). There were no identical sequences for 16S on GenBank. The relationships between *Euphrosinella* and *Euphrosinopsis* in the phylogenetic tree is unresolved (Fig. 5B). As COI sequencing was not successful in this study, all euphrosinid species are represented by only 16S and 18S, and in the case of the species from GenBank, only 16S.

##### Remarks.

CCZ species agrees well with the genus *Euphrosinopsis* in having five prostomial appendages, caruncle partially free from the body wall and the presence of large, deeply embedded eyes lateral to median antenna and caruncle. However, this species shows differences from all known species in this genus, suggesting it belongs to a new species. The presence of single, small, unbranched cirriform branchia per parapodium suggest affiliation with *E.crassiseta* and *E.horsti*, which share the same character. However, the new species differs from *E.crassiseta* in possessing the ringent chaetae and lacking the coarse serration on neurochaetae. The most similar species, *E.horsti* can be easily separated by having anal cirri fused instead of typical cylindrical tube feet as in the new species. For comparison with another new *Euphrosinopsis* species also described in this study see the remarks section for *E.ahearni* sp. nov.

##### Distribution.

Central Pacific Ocean, Eastern CCZ, the exploration areas UK-1 and NORI-D (Fig. 1).

##### Etymology.

This species is named for Preben Hall, the captain onboard the ship Maersk Launcher that was used in NORI-D expeditions in 2020 and 2021.

## ﻿Discussion

This study has added six annelid species, three of those formally described and one likely new, and 41 records to the knowledge of the benthic annelid macrofauna of the CCZ, bringing a published record from the targeted areas (Fig. 1) to 54 annelid species, with 19 of them formalised (see also Wiklund et al. 2019; Drennan et al. 2021).

Unlike other annelid taxa, each Amphinomida species is represented by several specimens (no singletons), most with wide CCZ-distribution. More importantly molecular data confirmed a wide abyssal distribution for one species identified as Bathychloeiacf.sibogae. This species has been found in CCZ (Central Pacific) and off Australia (South Pacific) with the sampling sites separated by the distance of ca. 7500 km. However, both sampling areas were at similar depths of ca. 4000 m, providing further evidence that genetic connectivity over large geographic areas is more likely to be maintained at similar depths (Taylor and Roterman 2017). Traditionally annelids have been assumed to have wide geographical ranges due to their potential for wide larval dispersal. However, this paradigm has been challenged by molecular studies, which have often revealed the presence of several, sometimes cryptic, species for annelids in general (see Nygren 2014 for details), including families targeted in this study (e.g., Barroso et al. 2010; Borda et al. 2013). Thus, findings of wide geographical ranges for annelids with support of molecular data remain rare (e.g., Ahrens et al. 2013; Georgieva et al. 2015; Eilertsen et al. 2018; Kobayashi et al. 2018; Neal et al. 2018), likely as a result of undersampling within the vast abyssal realm, as well as the reflection of dispersal abilities of different species and presence or absence of barriers to dispersal.

Molecular phylogeny of the family Euphrosinidae has not been undertaken hitherto, as the number of taxa available on GenBank is very low. The difficulties of getting COI from members of Euphrosinidae further complicates the analyses, and more data (both in terms of number of genetic markers and taxa,) is needed to resolve the relationships within this family. Our phylogenetic results (Fig. 5B) suggest a sister taxon relationship between *Euphrosinellageorgievae* sp. nov. and Antarctic specimens identified as Euphrosinellacf.cirratoformis (see also Brasier et al. 2016). Prior to this study, the genus *Euphrosinopsis* had a distribution restricted to the Antarctic waters (Kudenov 1993; Borda and Kudenov 2014), but CCZ has been found to harbour two *Euphrosinopsis* species, both new to science. A relationship between the deep-sea and Antarctic shelf fauna has been long been suggested and a continuity of the benthic fauna by means of the abyss has been proposed by some authors (e.g., Held 2000; Briggs 2003; Gage 2004; Clarke et al. 2005; Brandt et al. 2007; Strugnell et al. 2008). Due to the deeper than usual continental shelf, cold temperatures and at least seasonal darkness, the Antarctic shelf itself could be seen as an analogue to deep-sea environment. Other exclusively deep-sea annelid taxa have been previously found on the Antarctic shelf, e.g., the polynoid subfamily Macellicephalinae (Neal et al. 2012, 2017).

The phylogenetic analyses of the family Amphinomidae resulted in a tree similar to that of Borda et al. (2015), with the genera *Archinome*, *Chloeia*, and *Notopygos* falling into one strongly supported Archinominae clade (Fig. 5A). In this study, we have added the genus *Bathychloeia*, which formed a well-supported clade with the previously analysed Archinominae genera (Fig. 5A).

To summarise, the number of DNA sequences for benthic faunal groups from the CCZ available on GenBank are growing, representing echinoderms (e.g. Glover et al. 2016a; Christodoulou et al. 2020), cnidarians (Dahlgren et al. 2016), molluscs (Wiklund et al. 2017), annelids (Janssen et al. 2015; Bonifácio and Menot 2019; Wiklund et al. 2019; Guggolz et al. 2020; Drennan et al. 2021; Neal et al. 2022), poriferans (Lim et al. 2017) and crustaceans (e.g. Janssen et al. 2015; Kaiser et al. 2018; Bribiesca-Contreras et al. 2021; Mohrbeck et al. 2021). The information presented here therefore represents a further step in improving our understanding of the benthic fauna from the CCZ area, which in turn is essential for informing conservation efforts, as well as eventually providing practical identification guides to the fauna of this region facilitating any future assessments of biodiversity change as part of environmental monitoring programs.

## Supplementary Material

XML Treatment for Bathychloeia

XML Treatment for Bathychloeia cf. balloniformis

XML Treatment for Bathychloeia cf. sibogae

XML Treatment for Paramphinome

XML Treatment for Paramphinome

XML Treatment for Euphrosinella

XML Treatment for Euphrosinella georgievae

XML Treatment for Euphrosinopsis

XML Treatment for Euphrosinopsis ahearni

XML Treatment for Euphrosinopsis halli

## Supplementary materials

Supplementary material 1 DarwinCore database of CCZAmphinomida Data type excel file Explanation note DarwinCore database. https://binary.pensoft.net/file/786222This dataset is made available under the Open Database License (http://opendatacommons.org/licenses/odbl/1.0/). The Open Database License (ODbL) is a license agreement intended to allow users to freely share, modify, and use this Dataset while maintaining this same freedom for others, provided that the original source and author(s) are credited.Muriel Rabone

## References

[B1] Ahrens JB Borda E Barroso R Paiva PC Campbell AM Wolf A Nugues MM Rouse GW Schulze A (2013) The curious case of *Hermodicecarunculata* (Annelida: Amphinomidae): evidence for genetic homogeneity throughout the Atlantic Ocean and adjacent basins. Molecular Ecology 22(8): 2280–2291. 10.1111/mec.12263 23517352

[B2] Averincev VG (1972) Benthic polychaetes Errantia from the Antarctic and Subantarctic collected by the Soviet Antarctic Expedition]. Issledovaniya fauny morei. Zoologicheskii Institut Akademii Nauk USSR 11(19): 88–292. [Biological Results of the Soviet Antarctic Expeditions, 5]

[B3] Barroso R Paiva PC (2008) A new deep-sea species of *Paramphinome* (Polychaeta: Amphinomidae) from southern Brazil. Journal of the Marine Biological Association of the United Kingdom 88(4): 743–746. 10.1017/S0025315408001549

[B4] Barroso R Paiva PC (2011) A new deep-sea species of *Chloeia* (Polychaeta: Amphinomidae) from southern Brazil. Journal of the Marine Biological Association of the United Kingdom 91(2): 419–423. 10.1017/S0025315410001499

[B5] Barroso R Klautau M Solé-Cava AM Paiva PC (2010) *Eurythoecomplanata* (Polychaeta: Amphinomidae), thecosmopolitan’ fireworm, consists of at least three cryptic species. Marine Biology 157(1): 69–80. 10.1007/s00227-009-1296-9

[B6] Barroso R Kudenov JD Halanych KM Saeedi H Sumida PY Bernardino AF (2018) A new species of xylophilic fireworm (Annelida: Amphinomidae: Cryptonome) from deep-sea wood falls in the SW Atlantic. Deep-sea Research. Part I, Oceanographic Research Papers 137: 66–75. 10.1016/j.dsr.2018.05.005

[B7] Barroso R Kudenov JD Shimabukuro M Carrerette O Sumida PY Paiva PC Seixas VC (2021) Morphological, molecular and phylogenetic characterization of a new Chloeia (Annelida: Amphinomidae) from a pockmark field. Deep-sea Research. Part I, Oceanographic Research Papers 171: 103499. 10.1016/j.dsr.2021.103499

[B8] Beckers P Tilic E (2021) Fine structure of the brain in Amphinomida (Annelida). Acta Zoologica 102(4): 483–495. 10.1111/azo.12383

[B9] Bely AE Wray GA (2004) Molecular phylogeny of naidid worms (Annelida: Clitellata) based on cytochrome oxidase I. Molecular Phylogenetics and Evolution 30(1): 50–63. 10.1016/S1055-7903(03)00180-5 15022757

[B10] Blake JA (2016) *Kirkegaardia* (Polychaeta, Cirratulidae), new name for *Monticellina* Laubier, preoccupied in the Rhabdocoela, together with new records and descriptions of eight previously known and sixteen new species from the Atlantic, Pacific, and Southern Oceans. Zootaxa 4166(1): 1–93. 10.11646/zootaxa.4166.1.1 27701359

[B11] Blake JA (2017) PolychaetaOrbiniidae from Antarctica, the Southern Ocean, the abyssal Pacific Ocean, and off South America. Zootaxa 4218(1): 1–45. 10.11646/zootaxa.4218.1.1 28187682

[B12] Blake JA (2019) New species of Cirratulidae (Annelida, Polychaeta) from abyssal depths of the Clarion-Clipperton Fracture Zone, North Equatorial Pacific Ocean. Zootaxa 4629(2): 151–187. 10.11646/zootaxa.4629.2.1 31712518

[B13] Blake JA (2020) New species and records of deep-water Orbiniidae (Annelida, Polychaeta) from the Eastern Pacific continental slope, abyssal Pacific Ocean, and the South China Sea. Zootaxa 4730(1): 1–61. 10.11646/zootaxa.4730.1.1 32229835

[B14] Böggemann M (2009) Polychaetes (Annelida) of the abyssal SE Atlantic. Organisms, Diversity & Evolution 9(4–5): 251–428.

[B15] Bonifácio P Menot L (2019) New genera and species from the Equatorial Pacific provide phylogenetic insights into deep-sea Polynoidae (Annelida). Zoological Journal of the Linnean Society 185(3): 555–635. 10.1093/zoolinnean/zly063

[B16] Borda E Kudenov JD (2014) Euphrosinidae (Annelida: Amphinomida) collected from Antarctica (R/V Polarstern, 1984, 1986) with comments on the generic placement of *Euphrosinemagellanica* Ehlers, 1900. Proceedings of the Biological Society of Washington 126(4): 299–311. 10.2988/0006-324X-126.4.299

[B17] Borda E Kudenov JD Chevaldonné P Blake JA Desbruyères D Fabri MC Hourdez S Pleijel F Shank TM Wilson NG Schulze A (2013) Cryptic species of *Archinome* (Annelida: Amphinomida) from vents and seeps. Proceedings of the Royal Society B: Biological Sciences 280(1770): 20131876. 10.1098/rspb.2013.1876 PMC3779335 24026823

[B18] Borda E Yáñez‐Rivera B Ochoa GM Kudenov JD Sanchez‐Ortiz C Schulze A Rouse GW (2015) Revamping Amphinomidae (Annelida: Amphinomida), with the inclusion of *Notopygos*. Zoologica Scripta 44(3): 324–333. 10.1111/zsc.12099

[B19] Brandt A Gooday AJ Brandao SN Brix S Brökeland W Cedhagen T Choudhury M Cornelius N Danis B De Mesel I Diaz RJ Gillan DC Ebbe B Howe JA Janussen D Kaiser S Linse K Malyutina M Pawlowski J Raupach M Vanreusel A (2007) First insights into the biodiversity and biogeography of the Southern Ocean deep-sea. Nature 447(7142): 307–311. 10.1038/nature05827 17507981

[B20] Brasier MJ Wiklund H Neal L Jeffreys R Linse K Ruhl H Glover AG (2016) DNA barcoding uncovers cryptic diversity in 50% of deep-sea Antarctic polychaetes. Royal Society Open Science 3(11): 160432. 10.1098/rsos.160432 28018624 PMC5180122

[B21] Brenke N (2005) An epibenthic sledge for operations on marine soft bottom and bedrock. Marine Technology Society Journal 39(2): 10–21. 10.4031/002533205787444015

[B22] Bribiesca-Contreras G Dahlgren TG Drazen JC Drennan R Horton T Jones DO Leitner AB McQuaid K Smith CR Taboada S Wiklund H (2021) Biogeography and connectivity across habitat types and geographical scales in Pacific abyssal scavenging amphipods. Frontiers in Marine Science 8: 1028. 10.3389/fmars.2021.705237

[B23] Briggs JC (2003) Marine centres of origin as evolutionary engines. Journal of Biogeography 30(1): 1–18. 10.1046/j.1365-2699.2003.00810.x

[B24] Christodoulou M O’Hara T Hugall AF Khodami S Rodrigues CF Hilario A Vink A Martinez Arbizu P (2020) Unexpected high abyssal ophiuroid diversity in polymetallic nodule fields of the northeast Pacific Ocean and implications for conservation. Biogeosciences 17(7): 1845–1876. 10.5194/bg-17-1845-2020

[B25] Clarke A Barnes DK Hodgson DA (2005) How isolated is Antarctica? Trends in Ecology & Evolution 1(1): 1–3. 10.1016/j.tree.2004.10.004 16701330

[B26] Cohen BL Gawthrop A Cavalier–Smith T (1998) Molecular phylogeny of brachiopods and phoronids based on nuclear–encoded small subunit ribosomal RNA gene sequences. Philosophical Transactions of the Royal Society of London: Series B, Biological Sciences 353(1378): 2039–2061. 10.1098/rstb.1998.0351

[B27] Dahlgren TG Wiklund H Rabone M Amon DJ Ikebe C Watling L Smith CR Glover AG (2016) Abyssal fauna of the UK-1 polymetallic nodule exploration area, Clarion-Clipperton Zone, central Pacific Ocean: Cnidaria. Biodiversity Data Journal 9277: e9277. 10.3897/BDJ.4.e9277 PMC5018120 27660533

[B28] Dean HK (2008) The use of polychaetes (Annelida) as indicator species of marine pollution: A review. Revista de Biología Tropical 56(4): 11–38.

[B29] Detinova NN (1985) Polychaetous worms from the Reykjanes Ridge (the North AtlantiC). Bottom Fauna from Mid-Ocean Rises in the North Atlantic. Trudy Instituta okeanologii im. P.P. Shirshova 120: 96–136.

[B30] Donoghue MJ (1985) A critique of the biological species concept and recommendations for a phylogenetic alternative. The Bryologist 88(3): 172–181. 10.2307/3243026

[B31] Drennan R Wiklund H Rabone M Georgieva MN Dahlgren TG Glover AG (2021) *Neanthesgoodayi* sp. nov. (Annelida, Nereididae), a remarkable new annelid species living inside deep-sea polymetallic nodules. European Journal of Taxonomy 760: 160–185. 10.5852/ejt.2021.760.1447

[B32] Edgar RC (2004) MUSCLE: Multiple sequence alignment with high accuracy and high throughput. Nucleic Acids Research 32(5): 1792–1797. 10.1093/nar/gkh340 15034147 PMC390337

[B33] Eilertsen MH Georgieva MN Kongsrud JA Linse K Wiklund H Glover AG Rapp HT (2018) Genetic connectivity from the Arctic to the Antarctic: *Sclerolinumcontortum* and *Nicomachelokii* (Annelida) are both widespread in reducing environments. Scientific Reports 8(4810): 4810. 10.1038/s41598-018-23076-0 29556042 PMC5859262

[B34] Emson RH Young CM Paterson GLJ (1993) A fire worm with a sheltered life: Studies of *Benthoscolexcubanus* Hartman (Amphinomidae), an internal associate of the bathyal sea urchin *Archeopneusteshystrix* (A. Agassiz, 1880). Journal of Natural History 27(5): 1013–1028. 10.1080/00222939300770641

[B35] Fauchald K (1977) The polychaete worms, definitions and keys to the orders, families and genera. Natural History Museum of Los Angeles County: Los Angeles, CA (USA), Science Series 28: 1–188

[B36] Fauchald K Jumars PA (1979) The diet of worms: A study of polychaete feeding guilds. Oceanography and Marine Biology – an Annual Review 17: 193–284.

[B37] Fiege D Bock G (2009) A new species of *Archinome* (Polychaeta: Archinomidae) from hydrothermal vents on the Pacific-Antarctic Ridge 37 S. Marine Biological Association of the United Kingdom. Journal of the Marine Biological Association of the United Kingdom 89(4): 689–696. 10.1017/S0025315409000174

[B38] Folmer O Black M Hoeh W Lutz R Vrijenhoek R (1994) DNA primers for amplification of mitochondrial cytochrome c oxidase subunit I from diverse metazoan invertebrates 3: 294–299. 7881515

[B39] Gage JD (2004) Diversity in deep-sea benthic macrofauna: The importance of local ecology, the larger scale, history and the Antarctic. Deep-sea Research. Part II, Topical Studies in Oceanography 51(14–16): 1689–1708. 10.1016/j.dsr2.2004.07.013

[B40] Georgieva MN Wiklund H Bell JB Eilertsen MH Mills RA Little CT Glover AG (2015) A chemosynthetic weed: The tubeworm *Sclerolinumcontortum* is a bipolar, cosmopolitan species. BMC Evolutionary Biology 15(1): 1–7. 10.1186/s12862-015-0559-y 26667806 PMC4678467

[B41] Glover AG Smith CR Paterson GL Wilson GD Hawkins L Sheader M (2002) Polychaete species diversity in the central Pacific abyss: Local and regional patterns, and relationships with productivity. Marine Ecology Progress Series 240: 157–170. 10.3354/meps240157

[B42] Glover AG Wiklund H Rabone M Amon DJ Smith CR O’Hara T Mah CL Dahlgren TG (2016a) Abyssal fauna of the UK-1 polymetallic nodule exploration claim, Clarion-Clipperton Zone, central Pacific Ocean: Echinodermata. Biodiversity Data Journal (4). 10.3897/BDJ.4.e7251 PMC4759440 26929713

[B43] Glover AG Dahlgren TG Wiklund H Mohrbeck I Smith CR (2016b) An end-to-end DNA taxonomy methodology for benthic biodiversity survey in the Clarion-Clipperton Zone, central Pacific abyss. Journal of Marine Science and Engineering 4(1): 2. 10.3390/jmse4010002

[B44] Glover AG Wiklund H Chen C Dahlgren TG (2018) Point of view: Managing a sustainable deep-sea ‘blue economy’requires knowledge of what actually lives there. eLife 7: e41319. 10.7554/eLife.41319 PMC6257809 30479272

[B45] Gollner S Kaiser S Menzel L Jones DO Brown A Mestre NC Van Oevelen D Menot L Colaço A Canals M Cuvelier D Durden JM Gebruk A Egho GA Haeckel M Marcon Y Mevenkamp L Morato T Pham CK Purser A Sanchez-Vidal A Vanreusel A Vink A Martinez Arbizu P (2017) Resilience of benthic deep-sea fauna to mining activities. Marine Environmental Research 129: 76–101. 10.1016/j.marenvres.2017.04.010 28487161

[B46] Guggolz T Meißner K Schwentner M Dahlgren TG Wiklund H Bonifácio P Brandt A (2020) High diversity and pan-oceanic distribution of deep-sea polychaetes: *Prionospio* and *Aurospio* (Annelida: Spionidae) in the Atlantic and Pacific Ocean. Organisms, Diversity & Evolution 18(2): 1–7. 10.1007/s13127-020-00430-7

[B47] Gunton LM Neal L Gooday AJ Bett BJ Glover AG (2015) Benthic polychaete diversity patterns and community structure in the Whittard Canyon system and adjacent slope (NE Atlantic). Deep-sea Research. Part I, Oceanographic Research Papers 106: 42–54. 10.1016/j.dsr.2015.07.004

[B48] Gunton LM Kupriyanova EK Alvestad T Avery L Blake JA Biriukova O Böggemann M Borisova P Budaeva N Burghardt I Capa M Georgieva MN Glasby CJ Hsueh P-W Hutchings P Jimi N Kongsrud JA Langeneck J Meißner K Murray A Nikolic M Paxton H Ramos D Schulze A Sobczyk R Watson C Wiklund H Wilson RS Zhadan A Zhang J (2021) Annelids of the eastern Australian abyss collected by the 2017 RV ‘Investigator’voyage. ZooKeys 1020: 1–198. 10.3897/zookeys.1020.57921 33708002 PMC7930015

[B49] Hartman O (1959) Catalogue of the polychaetous annelids of the world. Part I. Allan Hancock Foundation Publications. Occasional Paper 23: 1–353.

[B50] Hartman O (1960) Systematic account of some marine invertebrate animals from the deep basins off southern California. Allan Hancock Pacific Expeditions 22(2): 69–216. [plates 1–19]

[B51] Hartmann-Schröder G Rosenfeldt P (1992) Die Polychaeten der “Polarstern”-Reise ANT V/1 in die Antarktis 1986. Teil 1: Euphrosinidae bis Iphitimidae. Mitteilungen aus dem Hamburgischen Zoologischen Museum und Institut 89: 85–124. [page(s): 87]

[B52] Held C (2000) Phylogeny and biogeography of Serolid Isopods (Crustacea, Isopoda, Serolidae) and the use of ribosomal expansion segments in molecular systematics. Molecular Phylogenetics and Evolution 15(2): 165–178. 10.1006/mpev.1999.0739 10837149

[B53] Horst R (1910) On the genus *Chloeia* with some new species from the Malay Archipelago, partly collected by the Siboga-Expedition. Notes from the Leyden Museum 32: 169–175.

[B54] Horst R (1912) Polychaeta errantia of the Siboga Expedition. Part 1, Amphinomidae. Siboga-Expeditie Uitkomsten op Zoologisch, Botanisch, Oceanographisch en Geologisch gebied verzameld in Nederlandsch Oost-Indië 1899-1900, 24a: 1–43 [10 plates] https://biodiversitylibrary.org/page/2187401 [page(s): 25]

[B55] Janssen A Kaiser S Meissner K Brenke N Menot L Martínez Arbizu P (2015) A Reverse Taxonomic Approach to Assess Macrofaunal Distribution Patterns in Abyssal Pacific Polymetallic Nodule Fields. PLoS ONE 10(2): e0117790. 10.1371/journal.pone.0117790 PMC4324633 25671322

[B56] Jeffreys RM Levin LA Lamont PA Woulds C Whitcraft CR Mendoza GF Wolff GA Cowie GL (2012) Living on the edge: Single-species dominance at the Pakistan oxygen minimum zone boundary. Marine Ecology Progress Series 470: 79–99. 10.3354/meps10019

[B57] Jumars PA Dorgan KM Lindsay SM (2015) Diet of worms emended: An update of polychaete feeding guilds. Annual Review of Marine Science 7(1): 497–520. 10.1146/annurev-marine-010814-020007 25251269

[B58] Kaiser S Brix S Kihara TC Janssen A Jennings RM (2018) Integrative species delimitation in the deep-sea genus *Thaumastosoma* Hessler, 1970 (Isopoda, Asellota, Nannoniscidae) reveals a new genus and species from the Atlantic and central Pacific abyss. Deep-sea Research. Part II, Topical Studies in Oceanography 148: 151–179. 10.1016/j.dsr2.2017.05.006

[B59] Katoh K Misawa K Kuma KI Miyata T (2002) MAFFT: A novel method for rapid multiple sequence alignment based on fast Fourier transform. Nucleic Acids Research 30(14): 3059–3066. 10.1093/nar/gkf436 12136088 PMC135756

[B60] Kearse M Moir R Wilson A Stones-Havas S Cheung M Sturrock S Buxton S Cooper A Markowitz S Duran C Thierer T Ashton B Meintjes P Drummond A (2012) Geneious Basic: An integrated and extendable desktop software platform for the organization and analysis of sequence data. Bioinformatics 28(12): 1647–1649. 10.1093/bioinformatics/bts199 22543367 PMC3371832

[B61] Kirkegaard JB (1995) Bathyal and abyssal polychaetes (errant species). Galathea Report 17: 7–56.

[B62] Kobayashi G Mukai R Alalykina I Miura T Kojima S (2018) Phylogeography of benthic invertebrates in deep waters: a case study of Sternaspiscf.williamsae (Annelida: Sternaspidae) from the northwestern Pacific Ocean. Deep-sea Research. Part II, Topical Studies in Oceanography 154: 159–166. 10.1016/j.dsr2.2017.12.016

[B63] Kudenov JD (1991) A new family and genus of the order Amphinomida (Polychaeta) from the Galapagos Hydrothermal vents. Ophelia, supplement 5 (Systematics, Biology and Morphology of World Polychaeta): 111–120.

[B64] Kudenov JD (1993) Amphinomidae and Euphrosinidae (Annelida: Polychaeta) principally from Antarctica, the Southern Ocean, and Subantarctic regions. Antarctic Research Series, Biology of the Antarctic Seas XXII 58: 93–150. 10.1029/AR058p0093

[B65] Lamarck JB (1818) [volume 5 of] Histoire naturelle des Animaux sans Vertèbres, préséntant les caractères généraux et particuliers de ces animaux, leur distribution, leurs classes, leurs familles, leurs genres, et la citation des principales espèces qui s’y rapportent; precedes d’une Introduction offrant la determination des caracteres essentiels de l’Animal, sa distinction du vegetal et desautres corps naturels, enfin, l’Exposition des Principes fondamentaux de la Zoologie. Paris, Deterville. Vol 5, 612 pp. http://biodiversitylibrary.org/page/12886879 [page(s): 327 [as ‘Amphinomae’]]

[B66] Lim SC Wiklund H Glover AG Dahlgren TG Tan KS (2017) A new genus and species of abyssal sponge commonly encrusting polymetallic nodules in the Clarion-Clipperton Zone, East Pacific Ocean. Systematics and Biodiversity 15(6): 507–519. 10.1080/14772000.2017.1358218

[B67] Lodge M Johnson D Le Gurun G Wengler M Weaver P Gunn V (2014) Seabed mining: International Seabed Authority environmental management plan for the Clarion–Clipperton Zone. A partnership approach. Marine Policy 49: 66–72. 10.1016/j.marpol.2014.04.006

[B68] Maciolek NJ (2020) *Anguillosyllis* (Annelida: Syllidae) from multiple deep-water locations in the northern and southern hemispheres. Zootaxa 4793(1): 1–73. 10.11646/zootaxa.4793.1.1 33056690

[B69] Maddison WP Maddison DR (2021) Mesquite: a modular system for evolutionary analysis. Version 3.70. http://www.mesquiteproject.org

[B70] McIntosh WC (1885) Report on the AnnelidaPolychaeta collected by H.M.S. Challenger during the years 1873–1876. Report on the Scientific Results of the Voyage of H.M.S. Challenger during the years 1873–76. Zoology (Jena, Germany) 12(part 34).

[B71] Medlin L Elwood H Stickel S Sogin M (1988) The characterization of enzymatically amplified eukaryotic 16S-like rRNA-coding regions. Gene 71(2): 491–499. 10.1016/0378-1119(88)90066-2 3224833

[B72] Mohrbeck I Horton T Jażdżewska AM Arbizu PM (2021) DNA barcoding and cryptic diversity of deep-sea scavenging amphipods in the Clarion-Clipperton Zone (Eastern Equatorial Pacific). Marine Biodiversity 51(2): 1–5. 10.1007/s12526-021-01170-3

[B73] Neal L Barnich R Wiklund H Glover AG (2012) A new genus and species of Polynoidae (Annelida, Polychaeta) from Pine Island Bay, Amundsen Sea, Southern Ocean – a region of high taxonomic novelty. Zootaxa 3542(1): 80–88. 10.11646/zootaxa.3542.1.4

[B74] Neal L Linse K Brasier MJ Sherlock E Glover AG (2017) Comparative marine biodiversity and depth zonation in the Southern Ocean: Evidence from a new large polychaete dataset from Scotia and Amundsen seas. Marine Biodiversity 48(1): 581–601. 10.1007/s12526-017-0735-y

[B75] Neal L Taboada S Woodall LC (2018) Slope-shelf faunal link and unreported diversity off Nova Scotia: Evidence from polychaete data. Deep-sea Research. Part I, Oceanographic Research Papers 138: 72–84. 10.1016/j.dsr.2018.07.003

[B76] Neal L Wiklund H Rabone M Dahlgren T Glover AG (2022) Abyssal fauna of polymetallic nodule exploration areas, eastern Clarion-Clipperton Zone, central Pacific Ocean: Annelida: Spionidae and Poecilochaetidae. Marine Biodiversity 52(51): 51. 10.1007/s12526-022-01277-1

[B77] Nygren A (2014) Cryptic polychaete diversity: A review. Zoologica Scripta 43(2): 172–183. 10.1111/zsc.12044

[B78] Nygren A Sundberg P (2003) Phylogeny and evolution of reproductive modes in Autolytinae (Syllidae, Annelida). Molecular Phylogenetics and Evolution 29(2): 235–249. 10.1016/S1055-7903(03)00095-2 13678679

[B79] Palumbi SR (1996) Nucleic acids II: the polymerase chain reaction. Molecular Systematics, 205–247.

[B80] Paterson GL Neal L Altamira I Soto EH Smith CR Menot L Billett DS Cunha MR Marchais-Laguionie C Glover AG (2016) New *Prionospio* and *Aurospio* species from the deep sea (Annelida: Polychaeta). Zootaxa 4092(1): 1–32. 10.11646/zootaxa.4092.1.1 27394364

[B81] Posada D (2008) jModelTest: Phylogenetic model averaging. Molecular Biology and Evolution 25(7): 1253–1256. 10.1093/molbev/msn083 18397919

[B82] Read G Fauchald K [Eds] (2021) World Polychaeta Database. Amphinomida. [Accessed through: World Register of Marine Species at:] http://www.marinespecies.org/aphia.php?p=taxdetails&id=893 [2021-05-14]

[B83] Righi S Savioli M Prevedelli D Simonini R Malferrari D (2021a) Unravelling the ultrastructure and mineralogical composition of fireworm stinging bristles. Zoology 144: 125851. 10.1016/j.zool.2020.125851 33227649

[B84] Righi S Savioli M Prevedelli D Simonini R Malferrari D (2021b) Response to Tilic and Bartolomaeus’s Commentary on the original Research Paper “Unravelling the ultrastructure and mineralogical composition of fireworm stinging bristles”(Zoology, 144). Zoology 144: 125889. 10.1016/j.zool.2020.125889 33454148

[B85] Ronquist F Teslenko M Van Der Mark P Ayres DL Darling A Höhna S Larget B Liu L Suchard MA Huelsenbeck JP (2012) MrBayes 3.2: Efficient Bayesian phylogenetic inference and model choice across a large model space. Systematic Biology 61(3): 539–542. 10.1093/sysbio/sys029 22357727 PMC3329765

[B86] Rouse GW Fauchald K (1997) Cladistics and polychaetes. Zoologica Scripta 26(2): 139–204. 10.1111/j.1463-6409.1997.tb00412.x

[B87] Sars GO (1872) On some remarkable forms of animal life from the great deeps off the Norwegian coast. Part 1, partly from posthumous manuscripts of the late prof. Mich. Sars. University Program for the 1^st^ half-year 1869. Brøgger & Christie, Christiania viii + 82 pp. [pls 1–6] http://biodiversitylibrary.org/page/11677777 [page(s): 45–49, pl. 4]

[B88] Sjölin E Erséus C Källersjö M (2005) Phylogeny of Tubificidae (Annelida, Clitellata) based on mitochondrial and nuclear sequence data. Molecular Phylogenetics and Evolution 35(2): 431–441. 10.1016/j.ympev.2004.12.018 15804413

[B89] Smith CR De Leo FC Bernardino AF Sweetman AK Arbizu PM (2008) Abyssal food limitation, ecosystem structure and climate change. Trends in Ecology & Evolution 23(9): 518–528. 10.1016/j.tree.2008.05.002 18584909

[B90] Smith CR Paterson G Lambshead J Glover A Rogers A Gooday A Kitazato H Sibuet M Galeron J Menot L (2011) Biodiversity, species ranges, and gene flow in the abyssal Pacific nodule province: predicting and managing the impacts of deep seabed mining. ISA Technical Study No. 3, International Seabed Authority, Kingston, Jamaica. [ISBN: 978-976-95217-2-841]

[B91] Smith CR Clark MR Goetze E Glover AG Howell KL (2021) Biodiversity, Connectivity and Ecosystem Function Across the Clarion-Clipperton Zone: A Regional Synthesis for an Area Targeted for Nodule Mining. Frontiers in Marine Science 8: e797516. 10.3389/fmars.2021.797516

[B92] Strugnell JM Rogers AD Prodöhl PA Collins MA Allcock AL (2008) The thermohaline expressway: The Southern Ocean as a centre of origin for deep-sea octopuses. Cladistics 24(6): 1–8. 10.1111/j.1096-0031.2008.00234.x 34892888

[B93] Taylor ML Roterman CN (2017) Invertebrate population genetics across Earth’s largest habitat: The deep‐sea floor. Molecular Ecology 26(19): 4872–4896. 10.1111/mec.14237 28833857

[B94] Tilic E Bartolomaeus T (2021) Commentary on:“Unravelling the ultrastructure and mineralogical composition of fireworm stinging bristles” by Righi et al. 2020. Zoology 144: 125890. 10.1016/j.zool.2020.125890 33451887

[B95] Verdes A Simpson D Holford M (2018) Are fireworms venomous? Evidence for the convergent evolution of toxin homologs in three species of fireworms (Annelida, Amphinomidae). Genome Biology and Evolution 10(1): 249–268. 10.1093/gbe/evx279 29293976 PMC5778601

[B96] Washburn TW Jones DO Wei CL Smith CR (2021) Environmental heterogeneity throughout the Clarion-Clipperton zone and the potential representativity of the APEI network. Frontiers in Marine Science 8: e319. 10.3389/fmars.2021.661685

[B97] Wedding LM Friedlander AM Kittinger JN Watling L Gaines SD Bennett M Hardy SM Smith CR (2013) From principles to practice: a spatial approach to systematic conservation planning in the deep sea. Proceedings of the Royal Society B: Biological Sciences 280(1773): 20131684. 10.1098/rspb.2013.1684 PMC3826217 24197407

[B98] Weigert A Helm C Meyer M Nickel B Arendt D Hausdorf B Santos SR Halanych KM Purschke G Bleidorn C Struck TH (2014) Illuminating the base of the annelid tree using transcriptomics. Molecular Biology and Evolution 31(6): 1391–1401. 10.1093/molbev/msu080 24567512

[B99] Wiklund H Nygren A Pleijel F Sundberg P (2008) The phylogenetic relationships between Amphinomidae, Archinomidae and Euphrosinidae (Amphinomida: Aciculata: Polychaeta), inferred from molecular data. Marine Biological Association of the United Kingdom. Journal of the Marine Biological Association of the United Kingdom 88(3): 509–513. 10.1017/S0025315408000982

[B100] Wiklund H Taylor JD Dahlgren TG Todt C Ikebe C Rabone M Glover AG (2017) Abyssal fauna of the UK-1 polymetallic nodule exploration area, Clarion-Clipperton Zone, central Pacific Ocean: Mollusca. ZooKeys 1(707): 1–46. 10.3897/zookeys.707.13042 PMC5674146 29118626

[B101] Wiklund H Neal L Glover AG Drennan R Rabone M Dahlgren TG (2019) Abyssal fauna of polymetallic nodule exploration areas, eastern Clarion-Clipperton Zone, central Pacific Ocean: Annelida: Capitellidae, Opheliidae, Scalibregmatidae, and Travisiidae. ZooKeys 883: 1–82. 10.3897/zookeys.883.36193 31719773 PMC6828828

